# The administration of intranasal live attenuated influenza vaccine induces changes in the nasal microbiota and nasal epithelium gene expression profiles

**DOI:** 10.1186/s40168-015-0133-2

**Published:** 2015-12-15

**Authors:** Y. Tarabichi, K. Li, S. Hu, C. Nguyen, X. Wang, D. Elashoff, K. Saira, Bryan Frank, Monika Bihan, E. Ghedin, Barbara A. Methé, Jane C. Deng

**Affiliations:** Division of Pulmonary and Critical Care Medicine, Department of Medicine, David Geffen SOM at UCLA, 37-131 CHS, 10833 Le Conte Avenue, Los Angeles, CA 90095 USA; Department of Medicine, UCLA, Los Angeles, CA USA; Department of Medicine Statistics Core, UCLA, Los Angeles, CA USA; Department of Computational and Systems Biology, University of Pittsburgh School of Medicine, Pittsburgh, PA USA; Department of Human Genome Medicine, J. Craig Venter Institute, 9704 Medical Center Drive, Rockville, MD 20850 USA; Department of Microbial and Environmental Genomics, J. Craig Venter Institute, 9704 Medical Center Drive, Rockville, MD 20850 USA; Department of Biology, Center for Genomics and Systems Biology, Global Institute of Public Health, New York University, New York, NY USA; Present address: Infectious Disease Research, Southern Research Institute, Birmingham, AL USA

**Keywords:** Human microbiome, Nasal colonization, Influenza, Interferons, Antiviral immune response

## Abstract

**Background:**

Viral infections such as influenza have been shown to predispose hosts to increased colonization of the respiratory tract by pathogenic bacteria and secondary bacterial pneumonia. To examine how viral infections and host antiviral immune responses alter the upper respiratory microbiota, we analyzed nasal bacterial composition by 16S ribosomal RNA (rRNA) gene sequencing in healthy adults at baseline and at 1 to 2 weeks and 4 to 6 weeks following instillation of live attenuated influenza vaccine or intranasal sterile saline. A subset of these samples was submitted for microarray host gene expression profiling.

**Results:**

We found that live attenuated influenza vaccination led to significant changes in microbial community structure, diversity, and core taxonomic membership as well as increases in the relative abundances of *Staphylococcus* and *Bacteroides* genera (both *p* < 0.05). Hypergeometric testing for the enrichment of gene ontology terms in the vaccinated group reflected a robust up-regulation of type I and type II interferon-stimulated genes in the vaccinated group relative to controls. Translational murine studies showed that poly I:C administration did in fact permit greater nasal *Staphylococcus aureus* persistence, a response absent in interferon alpha/beta receptor deficient mice.

**Conclusions:**

Collectively, our findings demonstrate that although the human nasal bacterial community is heterogeneous and typically individually robust, activation of a type I interferon (IFN)-mediated antiviral response may foster the disproportionate emergence of potentially pathogenic species such as *S. aureus*.

**Trial registration:**

This study was registered with Clinicaltrials.gov on 11/3/15, NCT02597647.

**Electronic supplementary material:**

The online version of this article (doi:10.1186/s40168-015-0133-2) contains supplementary material, which is available to authorized users.

## Background

Secondary bacterial pneumonia has been implicated as the main cause of death during influenza pandemics, including the 1918 “Spanish” and 2009 novel H1N1 influenza A virus pandemics [1–3]. The worldwide death toll was estimated to be around 50 million in 1918 and 284,400 in 2009 [4, 5]. Even in countries with advanced medical systems, bacterial co-infection remained a component of as many as 30 % of cases of H1N1 infection [6], evident in 29 % of post-autopsy lungs in one series [3]. The organisms identified by culture-dependent methods were predominantly *Staphylococcus* and *Streptococcus* species, with relatively higher proportions of *Streptococcus pneumoniae* and *Staphylococcus aureus* specifically [3, 6, 7].

While the lower respiratory tract had classically been considered sterile, there is growing evidence that it harbors its own microbiota [8, 9] and that this niche is informed to some extent by the contents of the nasopharyngeal and oral compartments [10–14]. Colonization of the nares and pharynx with pathogenic bacteria appears to be an essential precursor to the lower respiratory tract and other invasive bacterial infections or co-infection [15–19]. In murine systems, colonization by potentially pathogenic bacteria such as *S. pneumoniae* also appears to be enhanced in the presence of type I interferon (IFN) [20]. Murine models have demonstrated that both type I and type II IFN-mediated host responses to influenza infection act to critically suppress the antibacterial response against pulmonary infection by both *S. pneumoniae* and *S. aureus* [21–27]. Whether alterations in nasal colonization patterns occur during viral infections in human beings was unknown. Presumably, such a virally mediated change may permit the emergence or overgrowth of potentially pathogenic bacteria as a precursor to secondary bacterial infection. Further understanding of such a process would be essential towards the development of new interventions or preventative modalities that might depend on tempering the adverse changes in the nasal microbiota and/or host response.

We therefore addressed two questions in this study: We examined (1) how a new viral stimulus affected the upper respiratory tract microbiota and (2) its impact on host immune response to observe if any associations existed between the host immune response to the intranasal live attenuated vaccine (LAIV) and changes in bacterial composition. This nasal spray vaccine contains live, attenuated influenza viruses that are cold-adapted and temperature-sensitive, thereby preferentially replicating in the nares but not the lower respiratory tract. Live attenuated influenza vaccine is designed to induce an immune response that mimics the one generated by live influenza [28].

To determine how the nasal microbiota changes during the acute, early recovery, and late recovery periods after viral perturbation, we analyzed serial nasal samples from healthy volunteers who were experimentally inoculated with LAIV. In order to examine potential host interactions in the setting of viral perturbation, we profiled host gene expression by microarray analysis after LAIV administration with a focus on type I and type II interferon-stimulated genes. As *Staphylococcus* emerged as one of the genera whose relative abundance increased following LAIV administration, we then validated our observations with a murine model looking at the association between interferon induction and nasal persistence of *S. aureus*.

## Results

### Volunteer characteristics and samples analyzed

We recruited healthy adult subjects who were randomized to receive either nasal LAIV or sterile nasal saline spray. Their nares were sampled at baseline (visit 1), 1–2 weeks (visit 2), and 4–6 weeks (visit 3) after intervention. A total of 17 volunteers completed all three visits of the study, with 10 volunteers administered LAIV and 7 receiving saline nasal spray (control group). The baseline characteristics of these volunteers are presented in Table 1. Both groups were comparable in age and gender distributions. Most subjects reported either weekly or daily nose-picking, which has been associated with *S. aureus* nasal carriage [29].

**Table 1:** Characteristics of the volunteers

	Healthy volunteer	Healthy volunteer
	Saline controls (*n* = 7)	LAIV (*n* = 10)
Age, mean (range)	21.3 (18–25) years	23.8 (18–32) years
Gender (% female)	23.5 %	29.4 %
Student (%)	100 %	70 %
Nose-picking		
Never	14.3 %	20 %
Weekly	71.4 %	50 %
Daily	14.3 %	30 %

Values noted are means with ranges expressed for the age. In addition to that noted above, all volunteers had no recent smoking history, did not swim, predominantly showered rather than bathed, and used no probiotics or antibiotics within 90 days of enrollment

### Changes in nasal diversity following LAIV versus saline administration

To determine how the diversity of the nasal microbiota changes following intranasal influenza vaccine in comparison to the microbiota of volunteers having received only the nasal saline spray (control), we obtained serial nasal samples and sequenced hypervariable regions of the 16S ribosomal RNA (rRNA) gene amplified from the isolated bacterial DNA. We obtained median values of 5489 (*σ* = 1291) and 7495 (*σ* = 1524) high-quality sequence reads per sample, for the V3–V5 and V1–V3 regions, respectively [30, 31]. In general, the V3–V5 primers resulted in the identification of more taxonomic units when compared to the V1–V3 primers (data not shown), particularly in the groups receiving nasal influenza vaccine. As a result, we focused all LAIV and control group analyses on sequencing from V3–V5 regions.

**Fig. 1:**
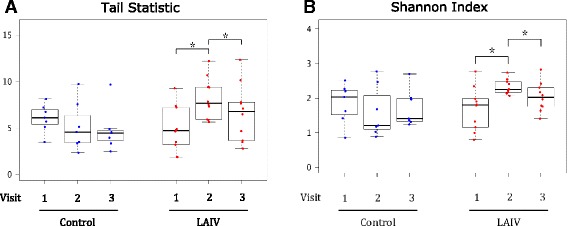
Measures of diversity in LAIV and control groups, by visit as ascertained by hypervariable regions V3–V5 sequencing. **a** demonstrates calculated tail statistics at all time points for either group (LAIV visit 1 to 2 *p* = 0.020; LAIV visit 2 to 3 *p* = 0.017). **b** demonstrates Shannon indices for all time points (LAIV visit 1 to 2 *p* = 0.014; LAIV visit 2 to 3 *p* = 0.049). (*p < 0.05 by Wilcoxon signed-rank testing)

At the genus level, taxonomic diversity was measured with the Shannon and Tail (τ) statistic [32] diversity indices, the latter being a more sensitive measure of diversity in the presence of low abundance taxa. Both the Tail statistic and the Shannon index demonstrated statistically significant within subject differences over time by two-way, repeated measures ANOVA (*p* = 0.047 and *p* = 0.032, respectively). Subsequent Wilcoxon signed-rank testing without correction for multiple testing revealed significant changes in both measures between the first and second visits and the second and third visits in the LAIV group, but no significant changes were seen in the control group (Fig. 1).

### Differences in community structure following LAIV versus saline administration

**Fig. 2:**
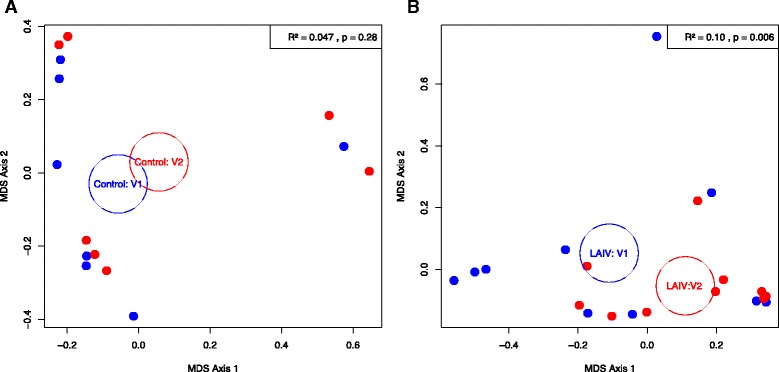
Multidimensional scaling plots demonstrating genus-level dissimilarities by Bray-Curtis indices between the first two visits in the control (**a**) and LAIV (**b**) groups. Data points from the first visits are in *blue*, while the second visits are in *red. Circles* represent respective centroid locations. V1 = visit 1, V2 = visit 2

We next sought to determine whether the community structures of either group did in fact change over time. In order to quantitatively represent these differences while accounting for inter-subject variability, we conducted a permutational multivariate analysis of variance (PERMANOVA) based on Bray-Curtis dissimilarities between all samples, stratified by subject. The interaction between the intervention (LAIV versus control) and time proved significant (*p* = 0.032), with a medium effect (*R*^2^ = 0.17). To study these differences within the individual groups, PERMANOVA was repeated for each group over the first two time points, when treatment-induced effects were predicted to be the greatest. The control group showed no significant changes in community structure over time (*p* = 0.28, *R*^2^ = 0.047), while the LAIV group changed significantly (*p* = 0.006, *R*^2^ = 0.10). These relationships were visually demonstrated by multidimensional scaling (MDS) plots based on the same dissimilarity measures (Fig. 2).

Within a cohort, microbiota variability may be attributed in a small part to sampling and sequencing variability, but more largely, to differences between individuals. Environmental conditions of a body habitat have been seen to limit microbiota variability; thus, a comparison of the microbiota’s variability between cohorts may provide information about changing conditions of a body habitat due to treatment. This variability can be measured by first quantifying the microbiome variance within each cohort and then testing for heteroscedasticity (i.e., non-constant variance) across cohorts. An analysis comparing the level of within group variability over time was performed based on the Bray-Curtis dissimilarities within each group (LAIV or control by visit), as measured by the average distance of samples from the centroid of each group. This analysis revealed a reduction in variability among the LAIV treated subjects at visits 2 and 3, compared to visit 1 (*p* < 0.0001 for both) (Additional file 1: Figure S1). The control group’s variance remained constant, i.e., was not heteroscedastic. This decrease in microbiome variability within the LAIV cohort further supports the notion that the LAIV treatment had an effect on the nasal habitat of the LAIV cohort. This may imply that conditions of the nasal habitat were changed by LAIV inoculation such that less variability in the microbiota was supportable.

### Genus and phylum level differences following LAIV versus saline control

We next examined how the nasal microbiota changed at the genus and phylum levels following LAIV versus saline administration. Analysis of the sequences for the V3–V5 regions revealed the presence of four prominent phyla (Actinobacteria, Firmicutes, Proteobacteria, and Bacteroidetes), which accounted for more than 90 % of all discovered taxa (Table 2). Over 75 % of the sequences mapped to 12 genera, 7 of which were ubiquitous in more than 95 % of the samples (*Corynebacterium*, *Propionibacterium*, Actinomycetales, *Staphylococcus*, *Streptococcus*, “Bacilli class,” and Bacillales). At baseline, the relative abundances of most of the major genera in either the LAIV or control groups were comparable in terms of the order, although the mean values differed, demonstrating inter-subject variability. *Corynebacterium* had the highest mean abundance, followed by *Staphylococcus*. The relative abundance of the major genera within individuals was similar between V1–V3 and V3–V5 regions. The only exception was *Moraxella*, which was unexpectedly detected in high abundance in samples from two individuals in the control group by V3–V5 sequencing, but not by V1–V3.

**Table 2:** Mean relative abundance of detected phyla and genera by group and visit

		Controls			LAIV		
Phylum		Visit 1 (%)	Visit 2 (%)	Visit 3 (%)	Visit 1 (%)	Visit 2 (%)	Visit 3 (%)
Genera (in *italics*)							
Actinobacteria		37.46	38.94	35.42	45.97	23.52	38.96
*Corynebacterium*		24.89	25.75	25.16	34.44	15.40	30.86
*Propionibacterium*		10.29	11.09	8.00	6.66	6.21	5.35
*Actinomycetales*		1.41	1.62	1.60	3.09	1.42	2.16
Firmicutes		32.18	25.56	41.56	40.71	51.34	44.99
*Staphylococcus*		16.14	12.79	25.28	19.04	26.37	24.53
*Streptococcus*		1.11	2.14	0.49	8.37	4.68	4.26
*Bacilli Class*		2.67	2.10	4.10	3.60	4.80	4.32
*Bacillales*		1.87	1.37	2.74	1.85	2.56	2.55
Proteobacteria		23.91	30.29	13.34	5.28	6.92	5.01
*Moraxella*		11.66	22.16	10.51	0.68	0.02	0.12
*Pseudomonas*		7.59	3.12	0.87	0.03	1.26	0.07
*Enterobacteriaceae*		0.92	1.20	0.18	3.29	1.69	0.47
Bacteroidetes		1.40	0.85	0.04	2.60	7.87	4.95
*Bacteroides*		0.00	0.00	0.01	0.00	6.26	4.13
Cyanobacteria		1.36	1.85	1.41	1.04	3.66	3.65
*Streptophyta*		1.21	1.79	1.40	1.03	3.58	0.88
Fusobacteria		0.042	0.023	3.88	0.61	1.78	1.17
Phylum cumulative		96.35	97.51	95.65	96.21	95.09	98.73
Genera cumulative		79.76	85.13	80.34	82.08	74.25	79.70

The above shown are average relative abundances of the 6 most abundant phyla and 12 most abundant genera by group based on V3–V5 sequencing. Phyla are listed on the left while their corresponding genera are indented beneath. All OTUs had a ubiquity of over 90 % except *Moraxella*, *Pseudomonas*, Enterobacteriaceae, and *Bacteroides. Bacteroides* was notably almost exclusively present in the LAIV groups on visits 2 and 3. Cumulative genera and phyla representation is noted at the bottom

**Fig. 3:**
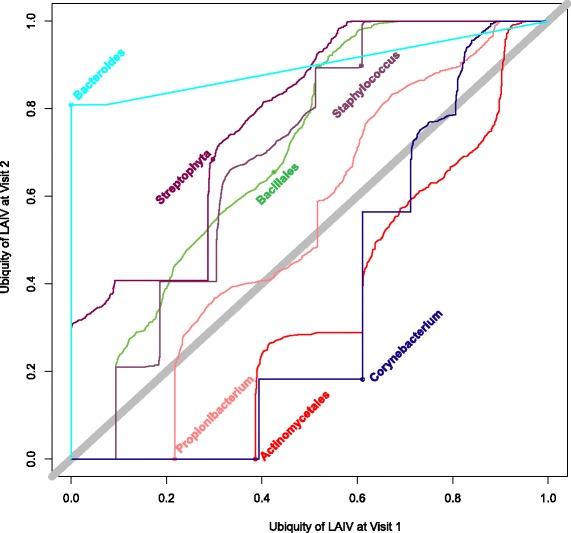
Ubiquity-ubiquity plot depicting the change in ubiquities for selected taxa using operational taxonomic units (OTUs) across the range of relative abundances between visits 1 and 2 in the LAIV group as ascertained by hypervariable regions V3–V5 sequencing. Only OTUs with a relative abundance greater than 0.5 % with a change in ubiquity greater than 20 % were included in this plot. OTUs that aligned to the right of the diagonal were more ubiquitous during visit 1, while those to the left were more ubiquitous at visit 2. The taxonomic classifications labeled are the closest, most confident taxonomic assignment that could be given to the OTU in question

Since we expected our interventions would have the highest effect at the first follow-up visit, we compared first visit and second visit samples on a taxon-by-taxon level utilizing a ubiquity-ubiquity (U-U) plot. The U-U plot visualizes how the ubiquity, i.e., the prevalence, of a taxon differs between the microbiotas of two groups at the same relative abundance threshold [33]. A curve is drawn for each taxon that represents any changes in ubiquity between the cohort depicted on the *y*-axis and the cohort on the *x*-axis, thereby identifying which taxa are most shifted between cohorts. Curves that fall closest to the thick gray diagonal line represent taxa for which the ubiquities are nearly identical for all abundances between the two cohorts. The U-U plot comparing the first and second visits of the LAIV and the control cohort with detectable levels (set to a threshold of 0.5 % relative abundance) of individual genera can be found in Fig. 3 and Additional file 2: Figure S2, respectively. We found that a higher percentage of subjects had *Bacteroides* and *Staphylococcus* present at visit 2 following LAIV administration. Interestingly, *Bacteroides* was undetectable in all subjects in the LAIV and control groups at baseline but reached a relative abundance of 10 % or more in 4/10 subjects in the LAIV group at visit 2. In addition, we identified the core taxa in each group by examining individual ubiquity-abundance (Ub-Ab) plots of each group of samples (Additional file 3: Figure S3). At a ubiquity threshold of 80 % and relative abundance threshold of 0.5 %, *Corynebacterium*, *Staphylococcus*, and *Propionibacterium* emerge as the core taxa at both time points in the control group and at the first visit in the LAIV group. The core taxa expanded by the second visit in the LAIV group to include *Staphylococcus*, *Streptococcus*, *Corynebacterium*, *Propionibacterium*, and Bacillales.

**Fig. 4:**
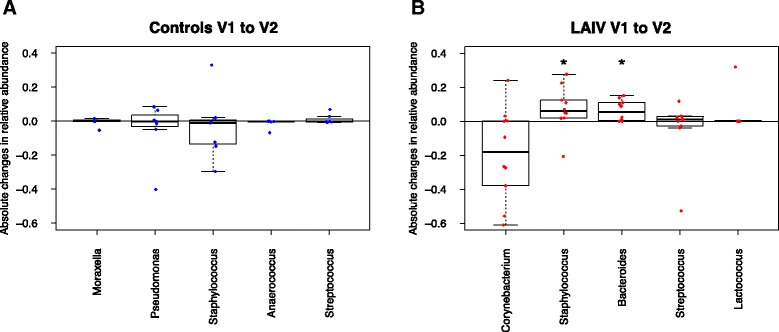
Absolute changes in relative abundance in the top five changed taxa in the control (**a**) and LAIV (**b**) groups as ascertained by hypervariable regions V3–V5 sequencing (in the LAIV group, *Staphylococcus p* = 0.048, *Bacteroides p* = 0.013). (*p < 0.05 by Wilcoxon signed-rank testing)

We next examined changes in the relative abundances of individual bacterial taxa, focusing on the most abundant bacterial taxa after LAIV administration (Fig. 4). We observe from the V3–V5 data absolute changes in the relative abundances of the genera that varied the most over time. While the control group displayed little change from baseline, the LAIV group had a notable decrease in the relative abundance of *Corynebacterium* and statistically significant increases in *Staphylococcus* and *Bacteroides* (*p* < 0.05 for both by Wilcoxon rank sum testing). Alternatively, such findings may also be explained by a reduction in other taxa and therefore may not represent an explicit increase in burden but could imply a greater relative representation.

Given that *Staphylococcus* species could cause invasive infections, we examined the temporal changes of this genus. At baseline, 4 out of the 7 subjects in the control group and 5 out of 10 subjects in the LAIV group had *Staphylococcus* detectable at a relative abundance above 10 %. Over time, the relative abundance of *Staphylococcus* in these individuals remained high (>10 %). Among the individuals who began with a relatively lower baseline level of *Staphylococcus* (i.e., below 10 %), the 3 subjects in control group continually demonstrated low levels of *Staphylococcus* throughout the study. In contrast, all of the 5 subjects in the LAIV group who began with a relative abundance of *Staphylococcus* below 10 % demonstrated a relative increase in abundance after LAIV administration, which subsequently remained elevated during the study (range 7–33 % at visit 2, 13–46 % at visit 3; Additional file 4: Figure S4). Hence, it appears that there are intrinsic factors that govern *Staphylococcus* persistence in the nares at a relatively high level in certain individuals over time. However, in a subset of individuals who do not have relatively high baseline levels of *Staphylococcus*, LAIV administration may be associated with increased colonization and persistence of *Staphylococcus* species, although the individual species could not be distinguished with sufficient confidence through the 16S regions sequenced in this study.

### Correlations between different genera

To identify the potential for taxonomic relationships among the detected bacteria, a correlation analysis was performed across the most abundant genera. Given the compositional aspect of the relative abundance data, the data was necessarily transformed by using the additive log ratio (ALR) transformation in order to eliminate spurious correlations. This was performed by dividing each of the 15 most abundant genera by the sum of the remaining relative abundances of the non-top 15 genera, followed by the natural logarithm transform [34]. A correlation matrix was constructed for the most abundant genera, (Additional file 5: Table S1) based on the control and LAIV cohort. This revealed statistically significant (*α* = 0.05) positive correlations (measures using Pearson’s correlation coefficient, *ρ*) between *Propionibacterium* and *Staphylococcus* (*ρ* = 0.52) and *Dolosigranulum* and *Corynebacterium* (*ρ* = 0.69), and a negative correlation between *Dolosigranulum* and both *Streptophyta* (*ρ* = −0.52) and *Anaerococcus* (*ρ* = −0.49). However, when corrected for multiple testing, the correlations were no longer statistically significant, most likely due to the small sample size used in the analysis.

In addition, we obtained serial nasal samples from outpatients presenting to the clinic or our emergency department with acute flu-like illness (FL). These samples were not included in the larger study because only 6 out of the 19 samples could be determined by the respiratory virus panel to be influenza A positive. However, they represent an important analog of viral stimulus to the upper respiratory tract microbiota human response when compared to vaccination using the LAIV. A pair-wise correlation comparison between the three cohorts (control, LAIV, and FL) was not performed due to the small cohort sizes. However, to determine if the previously identified relationships detected in the control and LAIV cohort would still hold in this larger dataset, the correlation analysis was repeated, this time utilizing all three cohorts (control, LAIV, and FL). Analysis of the samples including the FL cohort was performed on V1–V3 data, for which we had the most complete sequencing. When all three cohorts were combined and the correlation analysis was repeated, the correlations between *Propionibacterium* and *Staphylococcus* were strengthened (*ρ* = 0.55). The positive relationship between *Dolosigranulum* and *Corynebacterium* weakened (*ρ* = 0.56 from *ρ* = 0.69), and the negative relationship between *Dolosigranulum* and *Streptophyta* (*ρ* = −0.21 from *ρ* = −0.52) and *Anaerococcus* (*ρ* = −0.02 from (*ρ* = −0.49) also faded. The combined analysis (Additional file 6: Table S2), also identified additional taxonomic relationships, albeit less strong (0.19 < |*ρ*|), that were statistically significant, especially if a correction for multiple testing were to be applied. In summary, the FL cohort showed small differences from both the LAIV and control group, but due to the small sample sizes of each cohort and the relatively large interpersonal variation of each individual’s microbiota, the effects did not appear to be statistically significant. However, collectively, these analyses determined consistently positive correlations between *Staphylococcus* and *Propionibacterium*, suggesting that the most abundant genera move in unison [34].

### LAIV induces changes in nasal epithelial gene expression profiles, promoting the expression of interferon types I and II stimulated genes

**Fig. 5:**
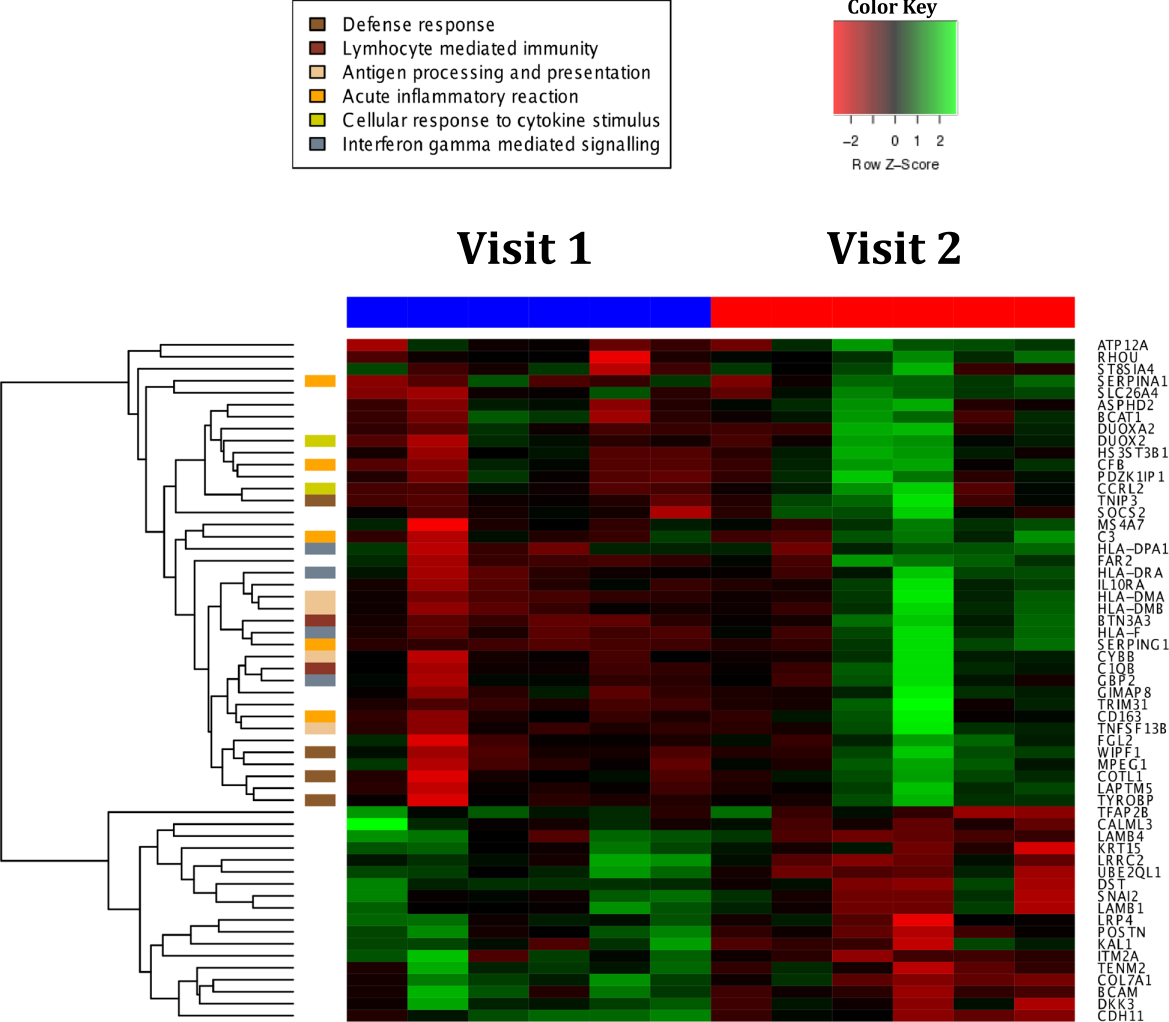
A heat map of log_2_ gene expression for the most differentially expressed genes in the LAIV group, selected by thresholds of *p* value <0.01 and log_2_ fold changes greater than 0.7 between visits. The *left hand column* shows selected gene ontology associations from the hypergeometric GO/BP analysis as described. Given significant overlap between gene ontology terms, representative groups were chosen to represent as many unique and non-overlapping GO/BP terms as possible

**Fig. 6:**
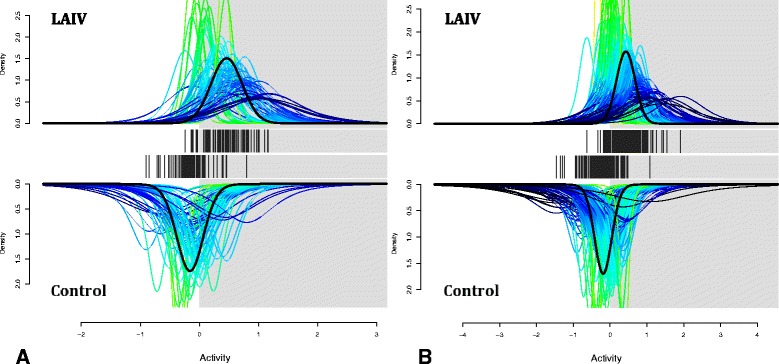
Gene set enrichment analysis for type I (**a**) and type II (**b**) interferon-stimulated genes in our expression sets. These figures demonstrate a probability density function for each gene, the center of which is marked by a vertical line on each *x*-axis. Distributions that lie predominantly to the right of the 0 mark are more likely up-regulated, while those to the left are not. Peaked and therefore narrower functions are represented by *green colors*, while broader distributions will tend towards the color *blue*. The sum of these functions, corrected for inter-gene correlation, is represented by a *thick black* distribution for each group. The LAIV group is shown on the upright graph, and the control group is drawn in the opposite direction for comparison. The LAIV group showed significant gene set enrichment in both types I and II interferon sets, when compared with the control group (*p* < 0.05)

To determine whether bacterial colonization might be influenced by host immune responses to LAIV, we concurrently sampled the nasal epithelium at each visit to examine host gene expression by microarray analysis. A total of 18 samples were obtained from 6 subjects in the LAIV group and 3 subjects in the control group concurrently with microbiota sampling (i.e., baseline and 1–2 weeks after LAIV or saline spray). Selecting for differentially expressed genes over time with absolute log_2_ fold change greater than 0.7 and *p* value less than 0.01 revealed 297 differentially expressed genes in the control group and 58 differentially expressed genes in the LAIV group. Hypergeometric testing for the enrichment of gene ontology (GO) [35, 36] terms in the up-regulated gene expression list for the LAIV group revealed 22 meaningful biological process, all related to immune responses and most related to antigen processing or lymphocyte activation (Fig. 5, Additional file 7: Table S3). The enriched GO terms in the control group, however, revealed only 8 biological processes that were non-specific, many of which involved ciliary function (Additional file 8: Table S4). Gene set enrichment testing, which was corrected for inter-gene correlation, showed a statistically significant up-regulation in both type I (Fig. 6a) and type II (Fig. 6b) interferon-stimulated genes (ISG) in the LAIV group when compared to expression in control group. The list of ISGs is provided in Additional file 9: Table S5.

#### Effect of type I IFN on persistence of *Staphylococcus aureus* in murine nasal model of colonization

**Fig. 7:**
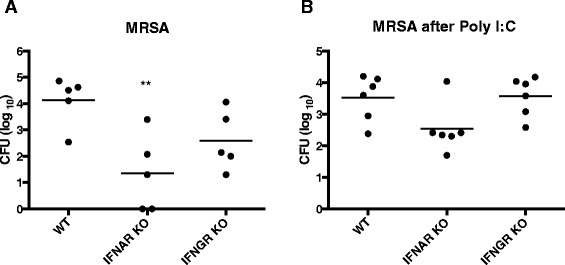
MRSA colonization is type I interferon-dependent. **a** Wild type (WT) C57Bl/6 mice, type I IFN receptor knockout (IFNAR KO), and type II IFN receptor knockout mice (IFNGR KO) underwent intranasal instillation of MRSA (6.7 × 10^7^ CFU). Twenty-four hours later, nasal lavage was performed using 200 μl of sterile saline for enumeration of CFU. **b** wild type, IFNAR, and IFNGR knockout mice were administered intranasal poly I:C (50 mcg) daily for 3 days, followed by i.n. MRSA (6.7 × 10^7^ CFU). Twenty-four hours later, nasal lavage was performed for enumeration of CFU. (**adjusted *p* < 0.01 by Kruskal-Wallis ANOVA with Dunn correction for multiple comparisons)

Given the robust induction of IFN signature genes following live attenuated influenza vaccine in the host transcriptional response, we sought to determine whether IFNs could enhance nasal colonization of *S. aureus*. We first established a mouse model of *S. aureus* nasal colonization (based upon work by Kiser, et al. [37]) but specifically used a methicillin-resistant strain of *S. aureus* given the epidemiologic importance of secondary MRSA infections following influenza. We found that wildtype (WT) mice develop more persistent *S. aureus* levels following intranasal instillation of MRSA compared to animals deficient in the type I IFN receptor (IFNAR KO, adjusted *p* = 0.008), with a trend towards decrease persistence of MRSA in animals deficient in type II IFN receptor (IFNGR KO, adjusted *p* = 0.14, Fig. 7a). To mimic the effects of LAIV on the interferon response, we next pretreated animals with intranasal poly I:C, which is a synthetic compound that mimics dsRNA present during viral infections, including influenza. We have previously shown that similar to influenza, poly I:C induces type I and type II IFN production, as well as downstream IFN-inducible genes such as IP-10 [38]. We examined whether MRSA persistence was dependent upon type I IFN, type II IFN, or both by administering intranasal poly I:C for 3 days to WT, IFNAR KO, and IFNGR KO animals, followed by intranasal MRSA instillation. We found that type I IFNs appear to promote the persistence of MRSA in the nares, as the animals deficient in type I IFN receptor had decreased bacterial burden (adjusted *p* = 0.08, Fig. 7b) at 24 hours compared to WT counterparts, whereas the absence of type II IFN had negligible effects on MRSA persistence. This suggests that type I interferons may contribute to the persistence of nasal *Staphylococcus* species, including MRSA.

## Discussion

Upper respiratory viral infections are frequently complicated by secondary bacterial infection [3, 6, 7]. Accordingly, we sought to determine whether an influenza virus infection might lead to changes in the nasal microbiota, thereby providing insights into interactions between host response and bacterial community structure. Previous studies include cross-sectional surveys of various upper respiratory samples from patients with pandemic H1N1 influenza infection [30, 39] and oropharyngeal samples from inpatients with acute viral infections [31]. These studies revealed no clear “post-viral signature microbiota” given the considerable heterogeneity in bacterial composition among individuals. In contrast, we obtained serial samples during viral perturbation to detect within subject changes in order to provide greater statistical power for our analyses. Additionally, our study is the first to compare dynamic changes of the nasal microbiota and host gene expression from baseline after an acute influenza challenge when compared to saline receiving controls. This design permitted us to contrast the magnitude of effect by a targeted viral stimulus against stochastic time-dependent differences. Despite our limited sample sizes, our approach allowed us to demonstrate small but significant differences in bacterial community structure and host immune response after viral perturbation. As anticipated, we observed great inter-subject heterogeneity of the nasal microbiota. Only upon accounting for inter-subject heterogeneity were we able to demonstrate significant, albeit small, changes in community structure with viral perturbation, based on Bray-Curtis similarities.

Similar to earlier studies, we observed a general preponderance of three dominant phyla—Actinobacteria, Firmicutes, and Proteobacteria—in all nasal samples [31, 39–45]. Core membership in the control group over time and the LAIV group prior to perturbation consisted of only three core genera—*Staphylococcus*, *Corynebacterium*, and *Propionibacterium*. The core membership expanded after LAIV administration to include *Streptococcus* and Bacillales, among others.

Notably, the number of subjects and our sequencing depth were greater than an earlier study examining the nasal microbiota pre- and post-rhinovirus experimental challenge [40], which enhanced our ability to detect significant differences in individual bacterial taxa. We were able to detect a significant increase in *Staphylococcus* relative abundance after LAIV administration. The difference in *Staphylococcus* abundance between the groups receiving saline or LAIV was particularly striking among subjects with low baseline levels of *Staphylococcus*. Although we were unable to confidently assign species of *Staphylococcus*, our animal data demonstrated that induction of an antiviral immune response promotes persistence of *S. aureus*. This supports a recent study where LAIV administration enhanced nasal colonization by MRSA and *S. pneumoniae* in a murine model [46]. Future studies will delve into the mechanisms by which *Staphylococcus* species may be permitted to outgrow other commensal bacteria in the upper respiratory tract.

Interestingly, we also observed the emergence of *Bacteroides*, an obligate anaerobe, after intervention in the LAIV group. While the absolute change was statistically significant in that group, the relative abundances were low, and the clinical relevance of this finding in the respiratory tract is not clear. However, these gram-negative microorganisms are typically noted to be anaerobic and associated with the gut microbiota where they can influence the host immune system and play both commensal and pathologenic ecological roles in part due to their diverse metabolic capabilities, including complex carbohydrate metabolism and biofilm formation [47]. It is possible that they may take on similar roles in the upper respiratory tract; thus, further study may be warranted.

Intertaxonomic bacterial interactions are of interest in determining how the microbiota is shaped within individuals. Of mounting interest in the study of the nasal microbiota is the determination of factors associated with the carriage of potentially pathogenic bacteria, including *S. pneumoniae* and *S. aureus*. Either of these organisms, when implicated in co-infection, leads to poorer clinical outcomes during influenza infection [2, 6, 19, 48, 49]. This link has prompted numerous investigators to seek associations or interactions between these pathogenic organisms and what may be considered “normal” commensals. Prior studies have reported an observation similar to ours, where the relative abundance of *Staphylococcus* is inversely proportional to the relative abundance of *Corynebacteria* [43, 50–52]. In support of this finding, Yan et al. demonstrated the inhibition of *S. aureus* by *C. pseudodiptheriticum* in vitro [52]. In parallel, Frank et al. [50] found that hospitalized patients had Firmicutes dominant profiles (relative abundance of 71 %) while the relative abundance of Actinobacteria proved smaller (20 %), supporting a pre-pathogenic potential in compromised hosts. This seemingly exclusive competition was also shown in a study by Uehara et al. [51] where a previously harvested strain of *Corynebacterium* was implanted in the nares of *S. aureus* carriers, leading to an eradication rate of 71 %, a result not replicated by saline or *S. epidermidis* implantation. However, given that microbiota data is compositional in nature, taxonomic correlations need to be estimated based on the (ALR) transformed abundances in order to avoid spurious correlations caused by scaling. Hence, our conclusions differ in that the three most abundant taxa (*Staphylococcus*, *Corynebacterium*, and *Propionibacterium*) appear to be positively correlated, which we believe reflects an overall stability in the bacterial community. These findings suggest that the factors that govern overall microbiota composition are in fact remarkably complex. Rather than focusing on individual bacterial proportions per se, perhaps a better grasp of the ecological characteristics of the nasal niche obtained via host and microbial metagenomic analysis might provide mechanistic insights into why certain bacteria persist in different individuals.

Since host immune response is one potential factor governing the changes in the bacterial composition, we obtained concurrent host response gene expression data pre- and post-LAIV administration. Despite their essential role in antiviral immunity, studies from our group and others have shown that the type I and II interferon responses are associated with suppression of the antibacterial component of the innate immune system in animal models of influenza and bacterial co-infections [21, 22, 25]. Moreover, type I IFNs are associated with enhanced colonization by *S. pneumoniae* in a murine model of nasal bacterial colonization [20]. Hence, we designed our study to determine whether experimental intranasal administration of LAIV would induce an IFN-mediated immune signature [53–55]. Hypergeometric gene set enrichment by conservative measures showed a statistically significant up-regulation in interferon types I and II stimulated genes in the LAIV group. Our results support a prominent local immune reaction in the nares after LAIV administration, as well as highlight a substantial role for ISG after the first week. Hence, we were able to confirm that our experimental intervention did lead to induction of an interferon immune signature at the time we saw the most significant changes in the nasal microbiota following LAIV. Furthermore, nasal administration of a viral ligand in a murine model led to increased persistence of MRSA in a type I interferon-dependent fashion. As LAIV has not been reported to predispose to secondary bacterial infection, it is likely that additional immune defects or perhaps structural changes in the respiratory epithelium induced by viral infection are necessary for the development of invasive disease. Nonetheless, increased colonization by potentially pathogenic bacteria may represent a critical first step.

Our study is not without limitations. The number of volunteers in each group was small; however, there were three samples obtained from each volunteer at different time points, allowing greater power with the benefit of paired statistical methods. Another limitation to our approach was an inability to make confident assignments of species to *Staphylococcus* operational taxonomic unit (OTUs) due to the less than 3 % difference in the V1–V3 regions between *S. aureus* and *S. epidermidis*. Finally, another limitation pertains to culture-independent 16S rRNA gene sequencing and that is the inability to distinguish live organisms from transient microorganism colonization, particularly with the potentially significant quantity of inhaled bacterial burden [56]. Additional repeated measures, i.e., increasing sample periodicity, from the same donor across all treatment cohorts, would improve our understanding of the rate of microbiota change and its potential variability and limits. This would provide a clearer relationship between donor treatment and cessation of symptoms versus the microbiota’s recovery towards “normality.” Identifying such lags might provide an avenue for better understanding cause and effect, instead of correlation alone.

## Conclusions

In summary, LAIV administration was associated with clinically meaningful changes in nasal epithelial gene expression that mirrored those expected of natural influenza infection, including activation of interferon-stimulated pathways, which have recently been implicated in diminished antibacterial activity and enhanced bacterial colonization in murine models. Along with these host gene expression changes, LAIV led to significant changes in bacterial community structure with increases in the relative abundance of *Staphylococcus* and *Bacteroides* genera. These changes support murine models and clinical findings, suggesting that they represent the first steps towards the emergence of potentially pathogenic bacteria during natural upper respiratory tract infection. Further investigation is needed to better appreciate these complex host-microbiota interactions in true disease states.

## Methods

### Enrollment

This study alternately assigned non-blinded cohorts of paid healthy adult volunteers to either LAIV: (FluMist, influenza vaccine, live; MedImmune) or saline nasal spray (Additional file 10: Figure S5). Volunteers were between the ages of 18 and 65 years and were without known medical problems. Any subjects with nasal ailments, on immunosuppressive medication (including nasal steroid spray or prednisone dose ≥20 mg or equivalent), exhibiting respiratory infection or antibiotic use within the last 90 days or with known chronic conditions were excluded. Upon volunteering, subjects were screened with a questionnaire that obtained general identifiers, contact information, and demographics such as age, sex, and ethnicity. Relevant data to the study included prior medical history, medication, or probiotic usage. Also addressed were the presence of habitual nose-picking and personal hygiene measures including frequency of bathing or showering, as well as swimming.

We also recruited adults who presented as outpatients to our emergency department or general internal medicine clinics with flu-like illness (FL), which we defined as having fever and symptoms of upper respiratory illness. The study was reviewed and approved beforehand by our human subjects institutional review board (UCLA IRB IRB#11-000326). The investigators received informed consent from all study participants.

### Sampling

Specimens were collected using sterile, dry swabs (EpiCentre). A total of four swabs (two from left nares, two from right) were obtained from each subject at enrollment and repeated at 1–2 weeks and then 4–6 weeks after initial presentation. In addition, subjects underwent nasal lavage with 10 ml of sterile non-microbiostatic saline at each time point. Following lavage, nasal brushings were obtained for extraction of host RNA for microarray analysis. For the FL group, we obtained serial samples during acute viral illness, 1–2 weeks later, and at 6–8 weeks after the initial presentation.

### Nucleotide extraction and pyrosequencing

The nasal swabs and lavage from each individual were pooled for isolation of bacterial genomic DNA (gDNA) using the PowerSoil DNA Isolation Kit (Mo-Bio laboratories). At the time of sample extraction, a negative control consisting of extraction reagents was included. V1–V3 and V3–V5 hypervariable regions of the 16S ribosomal RNA gene were amplified by PCR with barcoded primers designed using a set of algorithms developed at the J. Craig Venter Institute (JCVI) [57]. Prior to amplification, the PCRs were set up using precautions to reduce exogenous DNA contamination including use of a laminar flow hood and dispensing of reagents with pipettors restricted to PCR reagent use only (no gDNA).

Completed amplicons consisted of the following (per reaction): 2 μL of gDNA (~2–5 ng total), 0.75 units of Q5® High-Fidelity DNA Polymerase (New England Biolabs, MA) and 1× final concentration of Q5® High-Fidelity Master Mix. Primers were added to a final concentration of 200 nM, with dNTPs at a final concentration of 200 μM, along with the Q5 High GC Enhancer for amplification to a 1× final concentration and nuclease-free water to bring the final volume to 20 μL. PCR cycling conditions were as follows: initial denaturation of 30 s at 98 °C followed by 30 cycles of 98 °C for 10 s, 30 s at 56 °C, and 72 °C for 60 s, followed by a final incubation at 72 °C for 5 min before cooling to 4 °C. Negative controls (water blank) for PCR were also included. Negative controls (PCR and gDNA extraction controls) were subjected to an additional five cycles of PCR beyond the 30 cycles used for the experimental samples (35 cycles total). All PCR (2.5 μl from experimental samples, 10 μl from controls) were visualized on 1 % agarose gels. No visible bands of the expected amplicon size were detected from any of the negative controls.

Each reaction was cleaned individually using the Agencourt XP beads (Beckman Coulter, Inc., Indianapolis, IN) and resuspended in 30 μL of water prior to normalization and pooling of samples for sequencing. Amplicons were quantitated using the Quant-iT PicoGreen dsDNA Assay Kit (Life Technologies, Grand Island, NY) and then normalized amounts of each sample were combined into one pool prior to purification using a QIAQuick PCR Purification column (Qiagen, Valencia, CA) and submitted for sequencing with the Roche-454 FLX Titanium platform. The pooled samples were further cleaned using the Agencourt AMPure system (Beckman Coulter Genomics, Danvers, MA) prior to emulsification PCR (emPCR). Steps for emPCR, enrichment, and 454 sequencing were performed by following the vendor’s standard operating procedures. Real-time PCR was used to accurately estimate the number of molecules needed for emPCR. Total RNA was extracted from a subset of the specimens, and 18 samples were submitted for expression profile analysis by Affymetrix chip hybridization, following manufacturer recommendations [57].

#### Microbiota analysis

After sequencing, a read processing pipeline consisting of a set of custom perl scripts designed to efficiently format large datasets designed at the JCVI [32, 58] along with the mothur pipeline [59] were employed for deconvolution, trimming, and quality-filtering. Reads were first deconvoluted, i.e., assigned to samples based on their unique 10-nt barcode allowing no more than a one nucleotide mismatch to the barcode. After deconvolution, barcode and 16S primer sequences were removed allowing a maximum of six mismatches to the 16S primer and a maximum primer to barcode distance of 3 nt. The majority of primer matches (74.27 % perfect match + 22.23 % one mismatch = 96.5 %) of reads had one or fewer mismatches. Quality trimming of reads was performed using mothur. Reads with an average length of <100 nt and reads with “Ns” or with ambiguous base calls or a homopolymer longer than 8 nt were removed from subsequent analyses. A Blastn quality check was performed against an internal data set of 16S rRNA gene sequence reads to remove any sample reads not consistent with 16S rRNA gene sequences, in which at least 30 % of the query must be covered by the alignment (60 nt minimum). Passing reads were subsequently further processed including chimera checking through the mothur pipeline. After quality control processing, the high quality reads remaining were further examined using both taxonomy dependent (classification) and taxonomy independent (OTU formation) approaches. All steps through the generation of data matrices of taxonomic profiles were performed using the mothur pipeline. This includes generation of taxonomic classifications using the naïve Bayesian classification algorithm as implemented in mothur in conjunction with the SILVA database and formation of OTUs at 97 % sequence identity as well as subsequent taxonomic classification of the OTUs. Sequences for the project are publicly available at GenBank: PRJNA222379 [59].

### Distance-based analyses

Metric multidimensional scaling plots were generated with the help of the Vegan package in R (https://www.r-project.org/), with distances based on Bray-Curtis dissimilarities [60]. Inter-sample comparison with permutational multivariate analysis of variance (PERMANOVA) based on Bray-Curtis dissimilarities were performed using the Adonis function within the Vegan package. To control for inter-subject variability, the linear model utilized was distance = visit + treatment + visit × treatment + error, with stratification by subject. The factors under consideration were based on the treatment by visit interaction when more than two groups were analyzed, versus visit alone when a single group was analyzed. Results were reported based on bootstrapped *p* value and associated unadjusted *R*^2^. The dispersion analysis was performed by estimating the residuals for each sample by element-wise multiplying the estimated coefficients matrix (B_(*p* × *n*)_) by the transposed model matrix (X_(*n* × p)_)', where *p* is the number of regression coefficients estimated and *n* is the number of samples. With the resultant matrix, the absolute value of each column was summed, thus representing the total residuals associated with each sample. For each possible pair of groups, the median residual values underlying each group were compared for equality using the non-parametric Wilcoxon rank sum testing. *p* values were not corrected for multiple testing.

### Taxonomic abundance analyses

Normalized taxonomic abundance profiles were computed for each taxonomic category by dividing the number of reads assigned to a taxon by the total read count in each sample. The Shannon diversity index was calculated using Vegan, and the tail statistic, which is a rank-based diversity metric for analyzing low abundance taxa, was calculated as described by Li et al. [61]. Two-way, repeated measures ANOVA for the tail statistic and Shannon index were individually achieved in JMP version 11. The factors analyzed were visit (as a repeated measure) and study intervention.

For the correlation analysis between taxa, the (ALR) transform was performed by dividing each of the 15 most abundant genera by the sum of the relative abundances of the remaining non-top 15 genera. These ratios were then natural log transformed. To prevent performing the natural logarithm transformation on an abundance of 0, taxa with zero abundance were replaced with 1/10th of the lowest non-zero abundance across all samples and taxa. ALR transformed abundances are less prone to spurious correlation due to normalization since each transformed value is a ratio between its abundance and the reference “remaining” abundance. The top 15 taxa represented close to 92 % of the taxa by abundance.

### Corbata plots

Core microbiota analysis U-U plots were generated using Corbata [62]. Briefly, for each taxon in a group of samples, the relationship between abundance and ubiquity is calculated. Then, for each pair of groups, at matching abundances, a curve is generated to indicate the relative ubiquity of each taxon in each group. See the Material and Methods in Li 2013 [33] for a mathematically rigorous description of the algorithm. For interpretation, if groups A and B are represented on the *x*- and *y*-axis, respectively, if a taxon tends to be in greater ubiquity in group A then its curve will meander towards the bottom right of the plot (i.e., deviate from the diagonal line towards the *x*-axis). If the taxon tends to be in greater ubiquity in group B, then it will meander towards the top left of the plot or y-axis. When there is no difference between the ubiquity of a taxon between two groups, the curve will plot on the diagonal reference line. The Ub-Ab plot, also found in the Corbata suite of tools, represents the ubiquity of a taxon across all members of a cohort, as a function of abundance. Core taxa are defined as those taxa that are found in a majority of samples, at a significant abundance. Core taxa, which tend to be high in abundance and ubiquity can be identified by curves seeking the top right of the plot. While there are no standard ubiquity and abundance cutoffs, the 80 % ubiquity and 0.5 % abundance thresholds were chosen based upon their frequent ability to distinguish between the relatively few characteristic taxa for a body habitat and the remaining “background” taxa [33].

When the sample sizes are relatively small, U-U and Ub-Ab plot curves will be more jagged and step-shaped. Thus, smoothing was performed with bootstrapping by sampling with replacement of both the samples and reads during each bootstrap iteration. Also, frequently the U-U curve between two groups will fall along the diagonal, signifying that there is not a significant difference between the ubiquity of a taxon at all abundances between two cohorts. To improve the visualization, only taxa that were statistically significantly different using the Kolmogrov-Sminorv test were plotted.

### Microarray analysis

Extraction of RNA from nasal epithelial brushes was performed in the UCLA Clinical Microarray Core using the MagnaPure Compact automated system (Roche), which minimizes DNA contamination. RNA integrity was performed using the Eukaryote Total RNA Pico assay on the Agilent 2100 Bioanalyzer system. RNA quantification was determined by the NanoDrop 8000. For quality control assessment, the Affymetrix chips were inspected visually and felt to be free of gross spatial defects. More detailed analyses of the scanned data were obtained through the affyQCreport package of the Bioconductor project in R [63]. Intensity plots of individual chips were comparable, overlapping without outliers. Normalized unscaled standard error (NUSE) plots aligned across all chips. There was also the notable absence of significant variability between the percentage present and average background values in each chip. RNA degradation plots of each chip converged with similar slopes, suggesting agreement in the degrees of RNA digestion. As recommended by the manufacturer, all chips showed appropriate levels of expression for β-actin, though GADPH expression was just above the recommended level on three chips (two in the control group and one from the experimental group). Finally, inspection of the positive and negative border elements revealed positive and negative controls grouping in distinct intensity ranges, as expected.

Expression data was extracted using the affybatch package of the Bioconductor project in R. Background adjustment, normalization, and summarization were achieved across both sets independently (LAIV and control) using the well-established RMA or “robust multichip average” method [64–66]. Non-specific filtering was achieved with the nsFilter function, removing 50 % of genes with the lowest variance across all the chips, as well as gene IDs that did not match to an Entrez ID or matched to more than one Entrez ID. After filtering, the lmFit and eBayes functions were used to perform moderated paired *t* testing for each gene across visits 1 and 2.

At this level, gene set enrichment of ISG was performed with the QuSAGE package in R [67], comparing our processed gene lists against those from a prior study of A549 cell lines subjected to α or γ interferon [68].

Genes of interest from either group were then selected based on those with absolute log_2_ fold changes greater than 0.7 and individual *p* values less than 0.01. Both sets of up-regulated genes were subjected to conditional hypergeometric testing via the GOstats package to shed light on any potentially grouped biological processes [69]. Only the resultant processes with reported individual *p* values of less than 0.001 were reported. A heat map was generated from the gene list obtained from the LAIV arm using the heatmap.2 function in the gplots package, clustered by gene expression and with supervised labeling of up-regulated GO/BP functions based on prior hypergeometric testing.

### Animal model of nasal colonization

Age- and sex-matched mice with a C57BL/6 genetic background were used in all experiments. Wildtype (WT) C57BL/6, type I IFN receptor knockout (IFNAR KO) and type II IFN receptor knockout (IFNGR KO) animals were bred and housed in in-house colonies. For nasal colonization experiments, Los Angeles County (LAC) strain of methicillin-resistant *S. aureus* (MRSA) was used. Bacteria were grown overnight in tryptic soy broth in a shaking incubator at 37 °C, followed by 4-h subculture the following morning. An innoculum of 6 × 10^7^ colony-forming units (CFU) total was placed in the nares of each animal under anesthesia (intraperitoneal ketamine and xylazine (*n* = 5 animals/group). Poly I:C (50 μg in 50 μl) or sterile saline control was nasally instilled daily for 3 days prior to MRSA innoculation, where applicable (*n* = 6 animals/group). At 24 h after MRSA nasal instillation, animals were sacrificed for performance of nasal lavage with 200 μl of sterile saline to determine degree of persistence [70]. Bacterial CFU were enumerated by plating 10 μl of nasal lavage samples on TSA agar using 1:5 serial dilutions and back-calculated to determine the total number of CFU in nasal lavage fluid. All animal experiments were performed in accordance with NIH policies regarding the humane care and use of laboratory animals and were approved by the UCLA Office of Animal Research Oversight (OARO protocol 2005-143).

## Additional files

Additional file 1: Figure S1. Panel A demonstrates dispersion from the individual centroids for all groups across three visits based on Bray-Curtis dissimilarities in the aforementioned all-inclusive PERMANOVA model. Panel B shows resultant *p* values for each comparison between these groups using the Wilcoxon rank sum test. Red squares depict comparisons that are significant. V1 = visit 1, V2 = visit 2 and V3 = visit 3. Additional file 2: Figure S2. Ubiquity-ubiquity plot contrasting the ubiquities of a number of selected taxa, across a range of relative abundances between visits 1 and 2 in the control group as ascertained by hypervariable region V3–V5 sequencing. Only OTUs with a relative abundance greater than 0.5 % and change in ubiquity greater than 20 % were included in this plot. OTUs that aligned to the right of the diagonal were more ubiquitous during visit 1, while those to the left were more ubiquitous at visit 2. Additional file 3: Figure S3. Ubiquity-abundance plots for each group, by visit. These figures plot OTU ubiquities across a range of relative abundances, highlighting (with coloring and labels) OTUs at a threshold of at 80 % ubiquity and greater than 1 % relative abundance (represented by the red target) as ascertained by hypervariable region V3–V5 sequencing. This threshold reveals the “core” taxa. *Corynebacterium*, *Staphylococcus*, and *Propionibacterium* emerge as the core taxa at both time points in the control group and at the first visit in the LAIV group. The core taxa expanded by the second visit in the LAIV group to include, *Streptococcus* and Bacillales, among others. Additional file 4: Figure S4. Graphic representation of the relative abundance of *Staphylococcus* over time in subjects classified as having a low baseline level of *Staphylococcus* (i.e., below 10 %) at their first visit. The blue points represent the control group while the red points represent members of the LAIV group. Additional file 5: Table S1. Significant correlations among the most abundant genera for samples in the control and LAIV cohort. Pearson correlation coefficients (*ρ*) were calculated between the ALR transformed abundances for genera identified with the V1–V3 hypervariable regions of the 16S rDNA genes. Only genera with *p* values <0.05 (uncorrected for multiple testing) are provided below. The control and LAIV cohort used in this analysis consisted in total of 50 samples. Additional file 6: Table S2. Significant correlations among the most abundant genera with control, LAIV, and FL samples. Similarly to Table S1, Pearson correlation coefficients (*ρ*) were calculated between the ALR transformed abundances for genera identified with the V1–V3 regions. Only genera with *p* values <0.05 (uncorrected for multiple testing) were provided below. The control, LAIV, and flu-like cohort consisted in total of 107 samples. In support to the results shown in Table S1, the positive correlation between *Propionibacterium* and *Staphylococcus* and between *Dolosigranulum* and *Corynebacterium* seem to be reinforced with the addition of the FL cohort. Additional file 7: Table S3. Hypergeometric testing for the enrichment of GO/BP terms in the LAIV group. Additional file 8: Table S4. Hypergeometric testing for the enrichment of GO/BP terms in the control group. Additional file 9: Table S5. Type I and type II Interferon-stimulated gene list. Additional file 10: Figure S5. Flowchart of sampling procedures for subjects administered LAIV versus saline.

## Abbreviations

ALR: additive log ratio
FL: Flu-like illness cohort
GO/BP: gene ontology/biologic processes
ISG: interferon-stimulated genes
LAIV: live attenuated influenza vaccine
OTU: operational taxonomic unit
