# Supplementary figures and images for: The administration of intranasal live attenuated influenza vaccine induces changes in the nasal microbiota and nasal epithelium gene expression profiles
Source: Microbiome. 2015 Dec 15;3:74. doi: 10.1186/s40168-015-0133-2 (PMC4678663; doi:10.1186/s40168-015-0133-2)

A

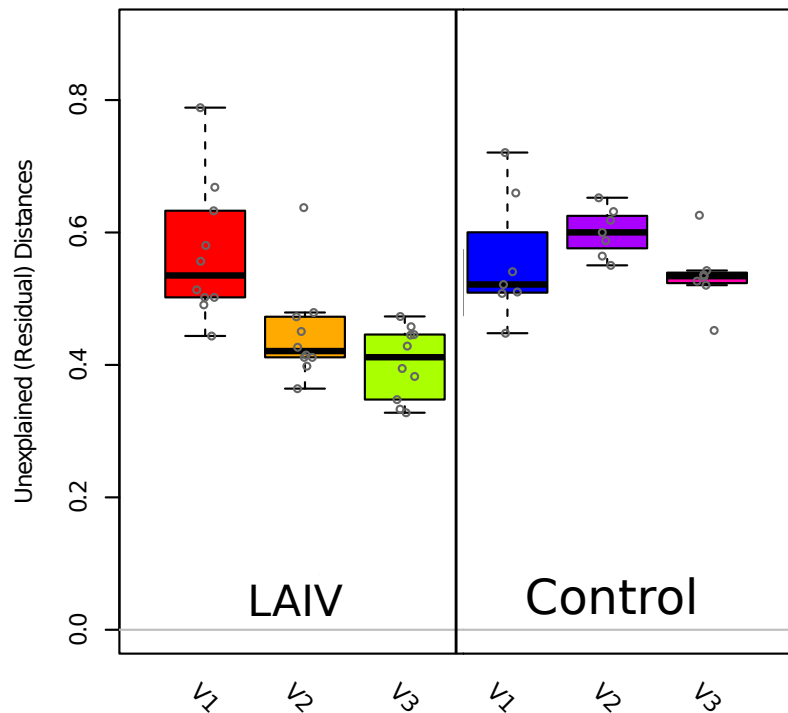

B

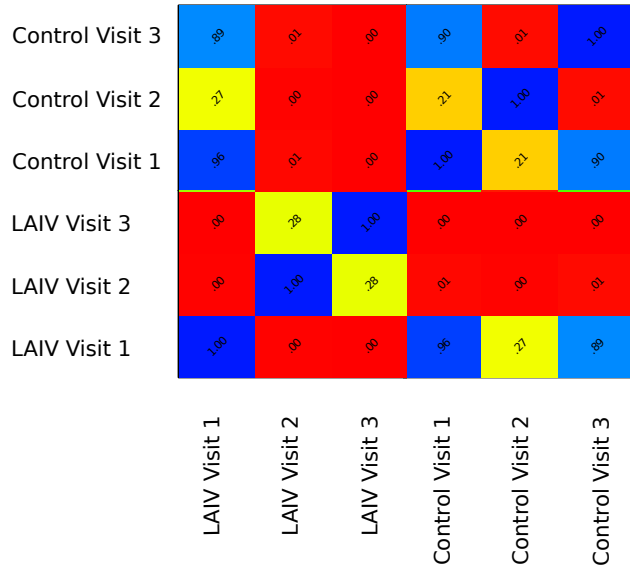

Supplement: Additional file 1: Figure S1. — Panel A demonstrates dispersion from the individual centroids for all groups across three visits based on Bray-Curtis dissimilarities in the aforementioned all-inclusive PERMANOVA model. Panel B shows resultant p values for each comparison between these groups using the Wilcoxon rank sum test. Red squares depict comparisons that are significant. V1 = visit 1, V2 = visit 2 and V3 = visit 3. [file 40168_2015_133_MOESM1_ESM.pdf]

Ubiquity of Controls at Visit 2

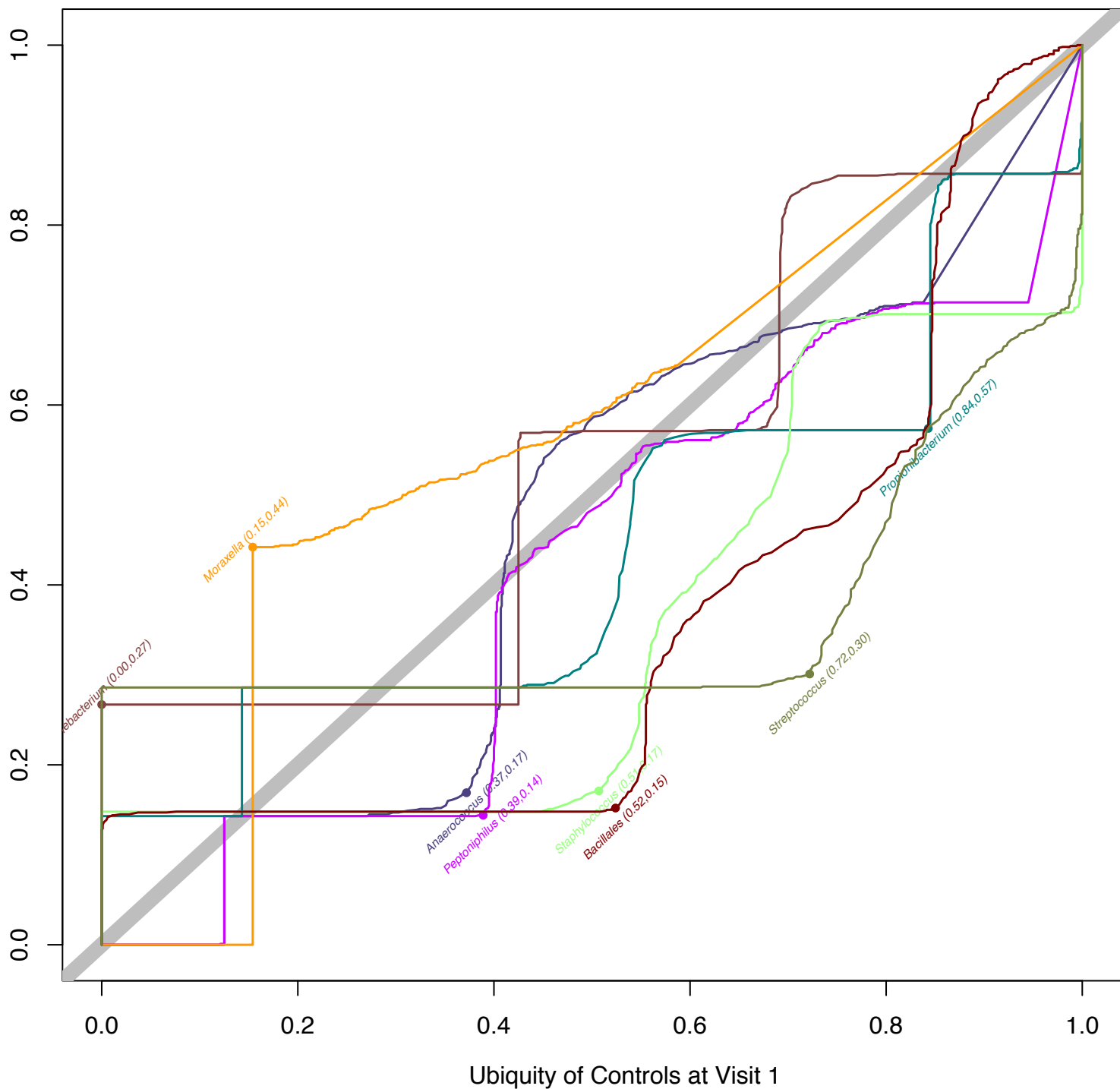

Supplement: Additional file 2: Figure S2. — Ubiquity-ubiquity plot contrasting the ubiquities of a number of selected taxa, across a range of relative abundances between visits 1 and 2 in the control group as ascertained by hypervariable region V3–V5 sequencing. Only OTUs with a relative abundance greater than 0.5 % and change in ubiquity greater than 20 % were included in this plot. OTUs that aligned to the right of the diagonal were more ubiquitous during visit 1, while those to the left were more ubiquitous at visit 2. [file 40168_2015_133_MOESM2_ESM.pdf]

**A** **Controls at Visit 1**

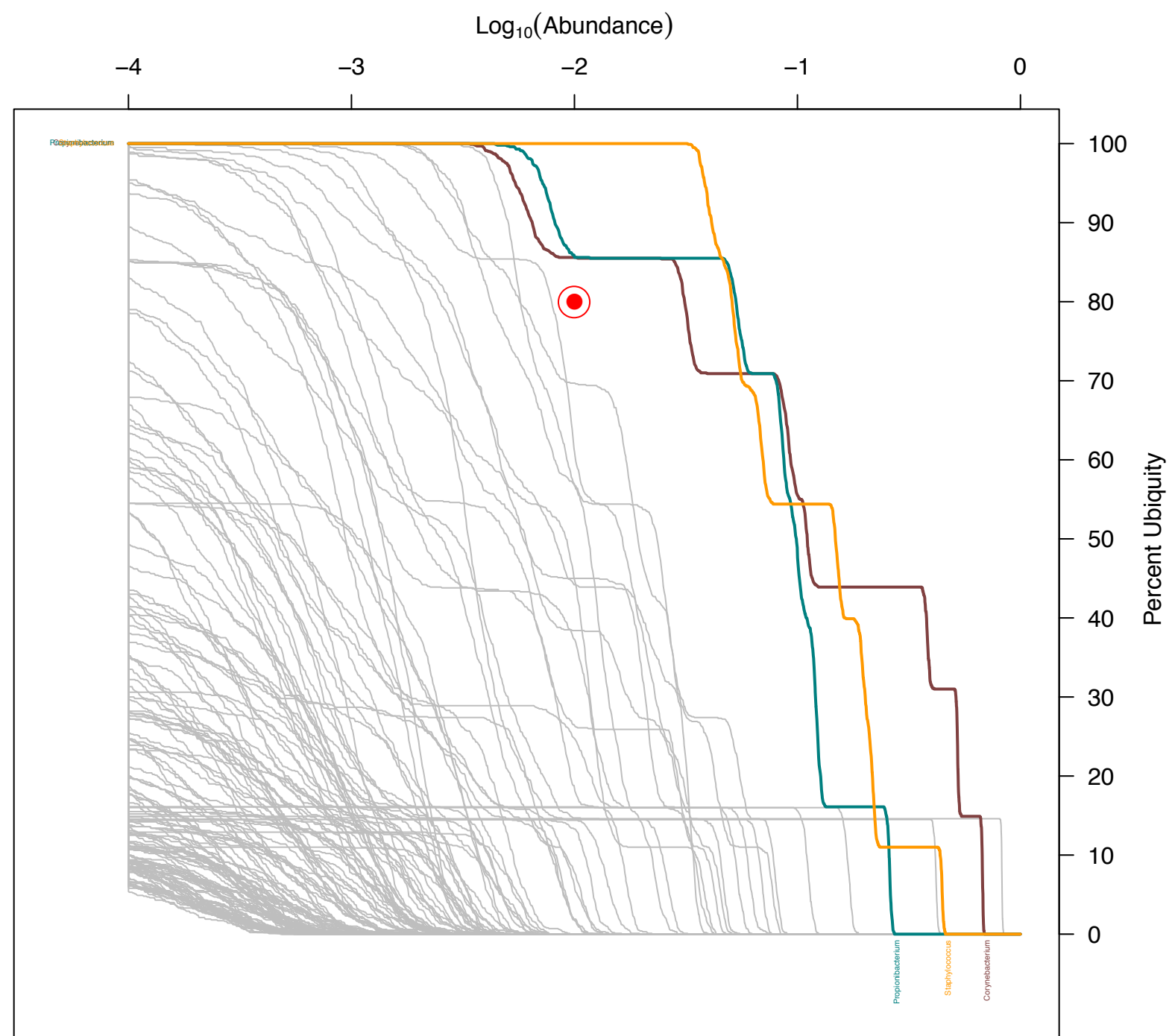

## B Controls at Visit 2

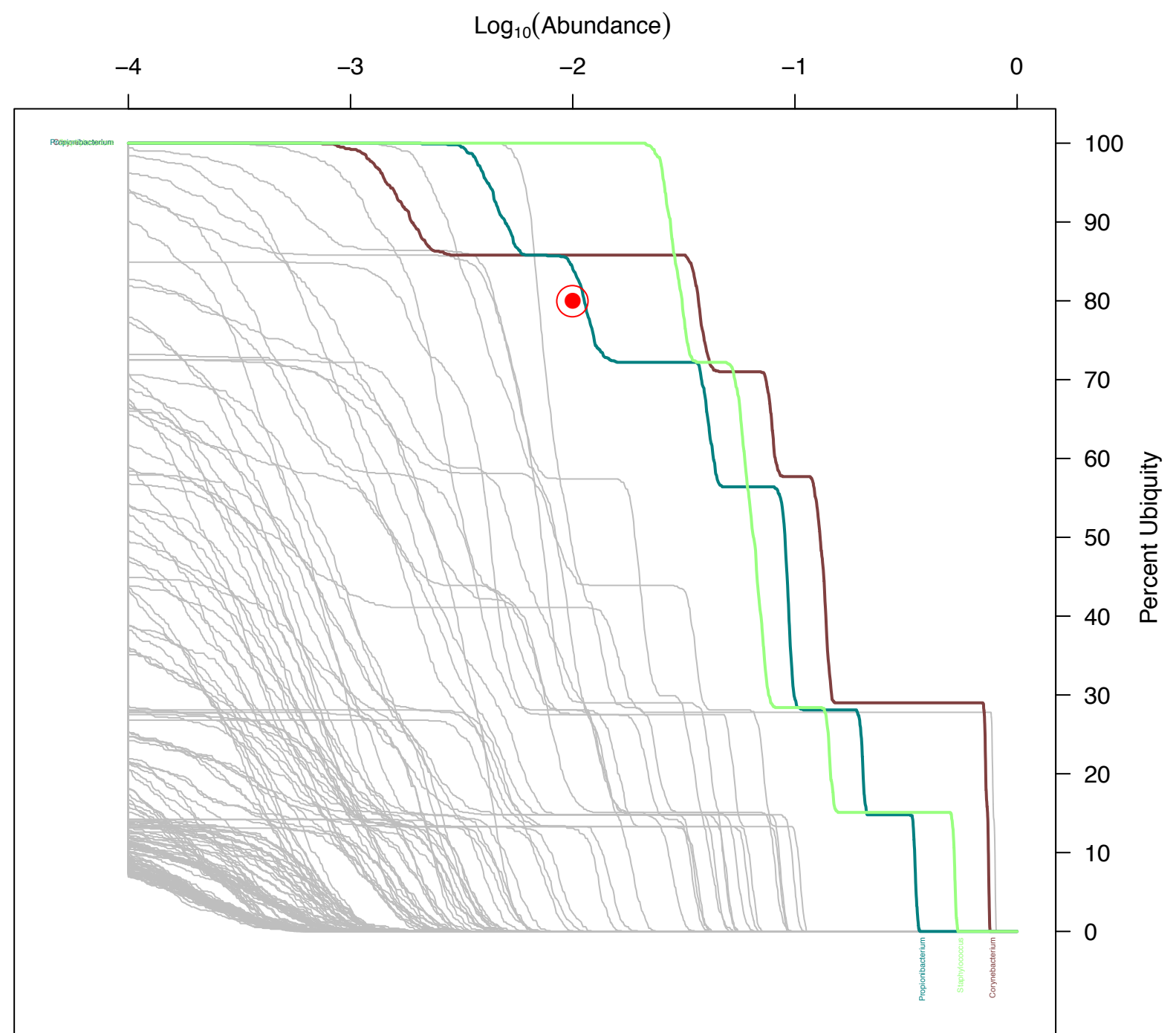

### C LAIV at Visit 1

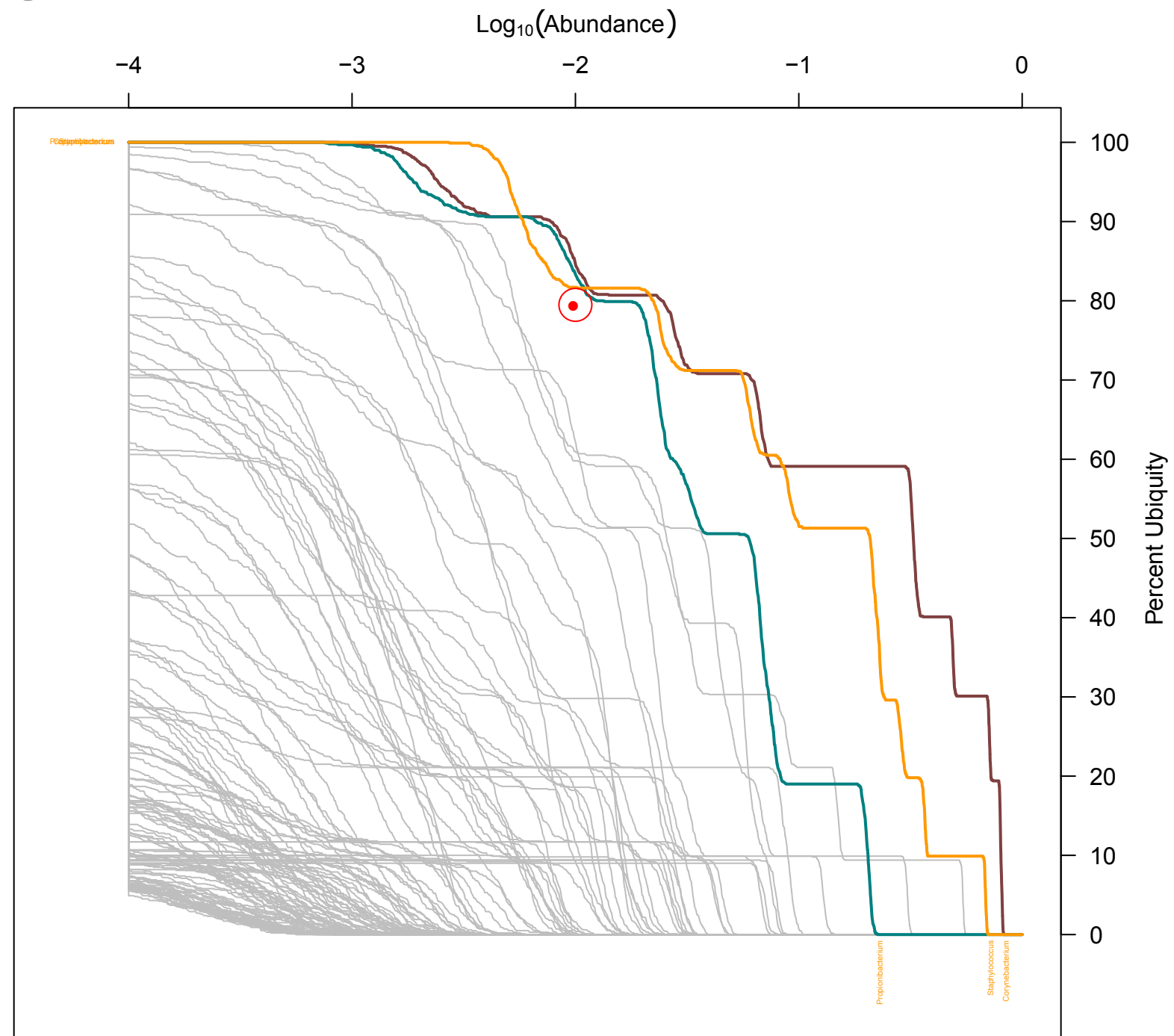

## D LAIV at Visit 2

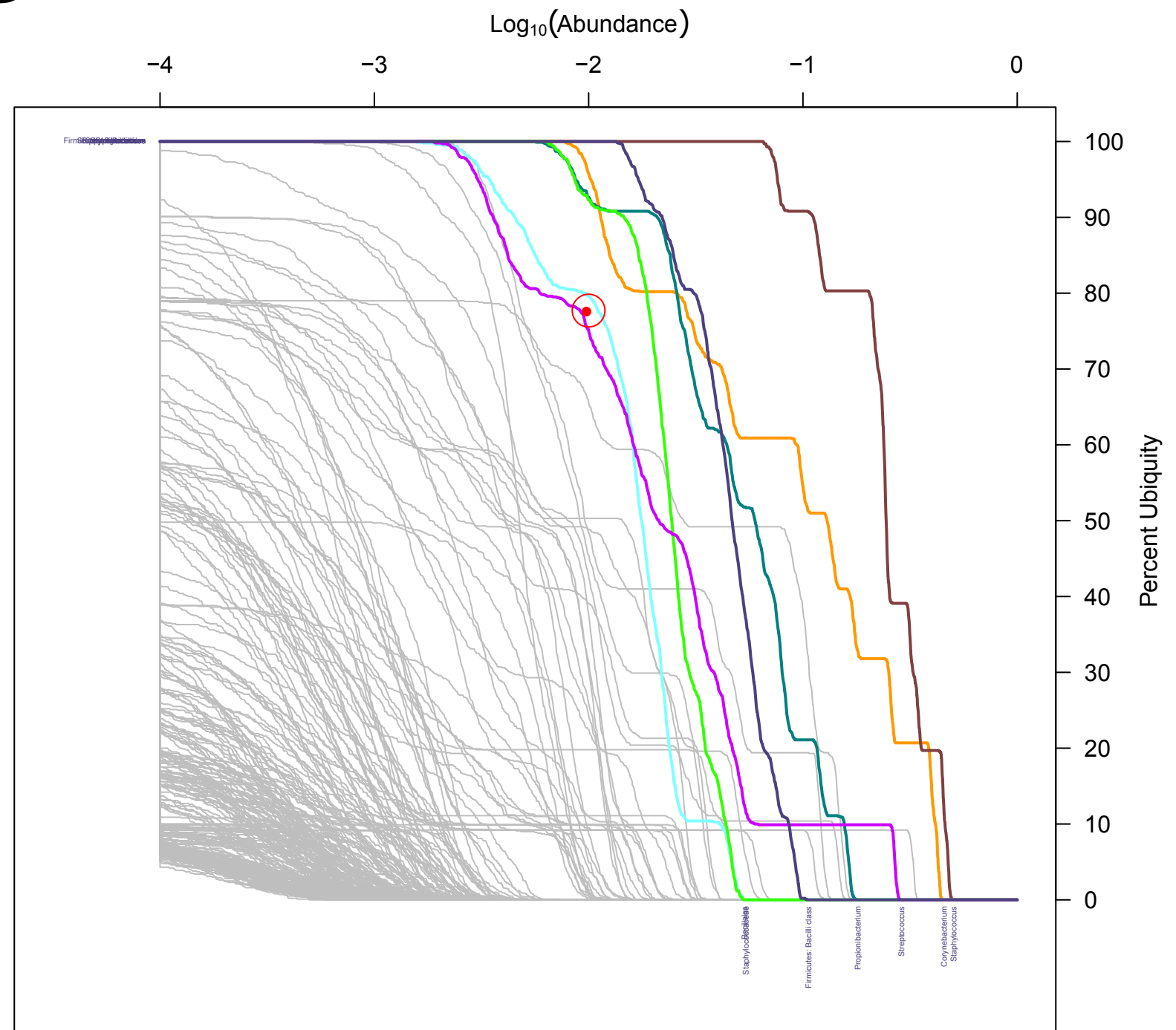

Supplement: Additional file 3: Figure S3. — Ubiquity-abundance plots for each group, by visit. These figures plot OTU ubiquities across a range of relative abundances, highlighting (with coloring and labels) OTUs at a threshold of at 80 % ubiquity and greater than 1 % relative abundance (represented by the red target) as ascertained by hypervariable region V3–V5 sequencing. This threshold reveals the “core” taxa. Corynebacterium, Staphylococcus, and Propionibacterium emerge as the core taxa at both time points in the control group and at the first visit in the LAIV group. The core taxa expanded by the second visit in the LAIV group to include, Streptococcus and Bacillales, among others. [file 40168_2015_133_MOESM3_ESM.pdf]

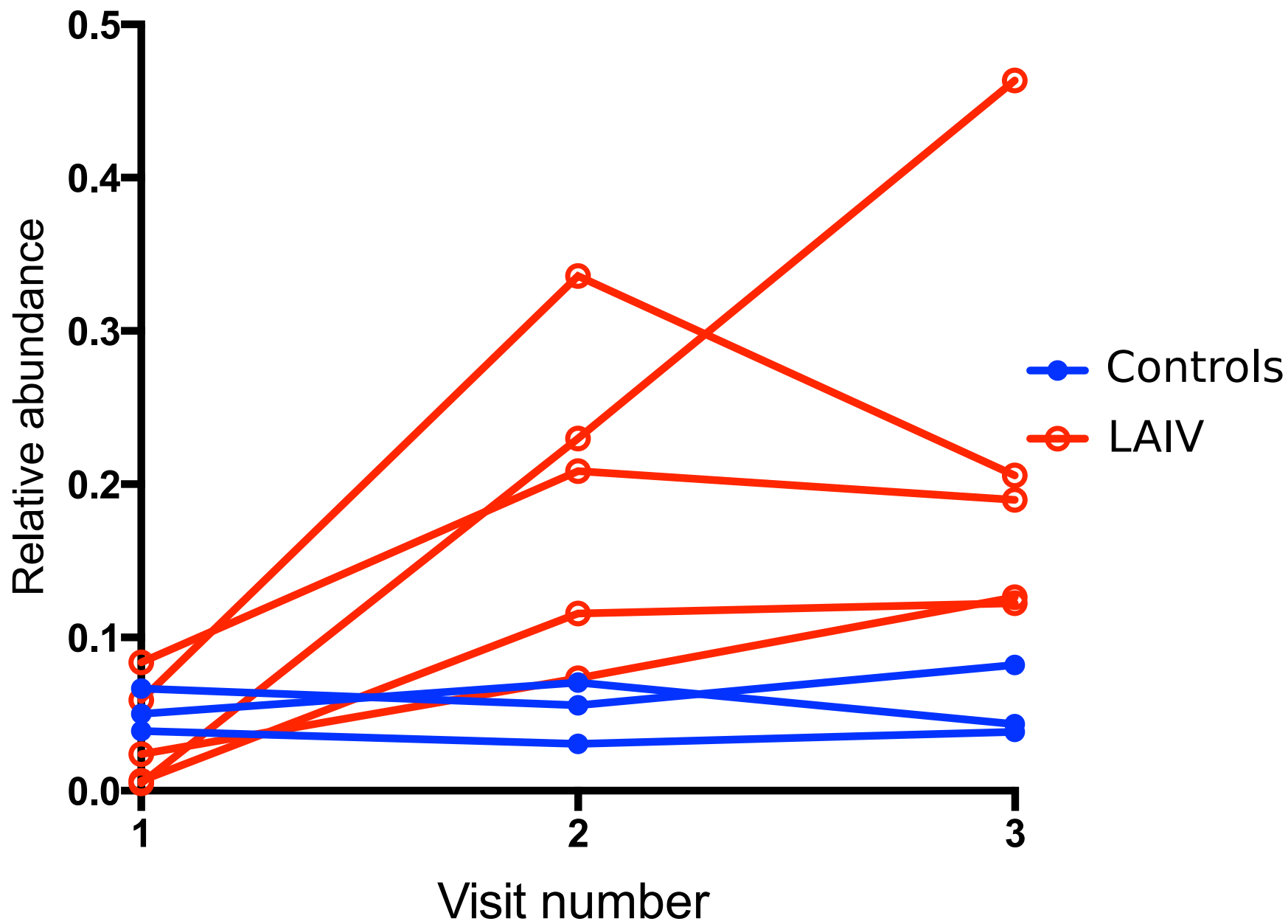

Supplement: Additional file 4: Figure S4. — Graphic representation of the relative abundance of Staphylococcus over time in subjects classified as having a low baseline level of Staphylococcus (i.e., below 10 %) at their first visit. The blue points represent the control group while the red points represent members of the LAIV group. [file 40168_2015_133_MOESM4_ESM.pdf]

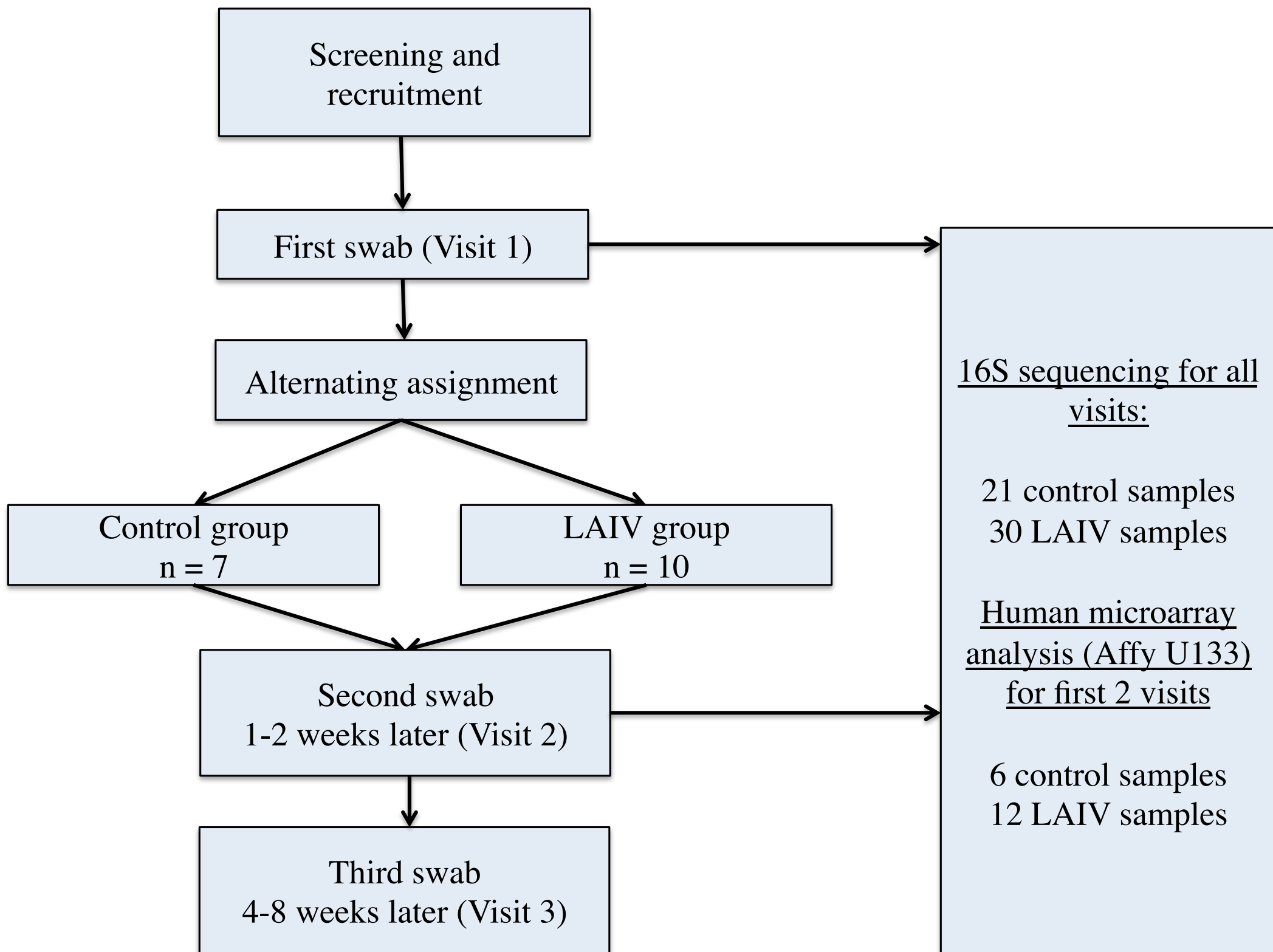

Supplement: Additional file 10: Figure S5. — Flowchart of sampling procedures for subjects administered LAIV versus saline. [file 40168_2015_133_MOESM10_ESM.pdf]
