# Supplementary material for: The administration of intranasal live attenuated influenza vaccine induces changes in the nasal microbiota and nasal epithelium gene expression profiles
Source: Microbiome. 2015 Dec 15;3:74. doi: 10.1186/s40168-015-0133-2 (PMC4678663; doi:10.1186/s40168-015-0133-2)
Supplement: Additional file 5: Table S1. — Significant correlations among the most abundant genera for samples in the control and LAIV cohort. Pearson correlation coefficients (ρ) were calculated between the ALR transformed abundances for genera identified with the V1–V3 hypervariable regions of the 16S rDNA genes. Only genera with p values <0.05 (uncorrected for multiple testing) are provided below. The control and LAIV cohort used in this analysis consisted in total of 50 samples. [file 40168_2015_133_MOESM5_ESM.docx]

Table S1. Significant correlations among the most abundant genera for samples in the Control and LAIV cohort. Pearson correlation coefficients (ρ) were calculated between the ALR transformed abundances for genera identified with the V1V3 hypervariable regions of the 16S rDNA genes. Only genera with p-values < 0.05 (uncorrected for multiple testing) are provided below. The control and LAIV cohort used in this analysis consisted in total of 50 samples.

|  |  |  |  |  |  |  |  |  | - | Anaerococcus |
| --- | --- | --- | --- | --- | --- | --- | --- | --- | --- | --- |
|  |  |  |  |  |  |  |  | - | - | Streptophyta |
|  |  |  |  |  |  |  | - | - | - | Haemophilus |
|  |  |  |  |  |  | - | - | - | - | Peptoniphilus |
|  |  |  |  |  | - | - | - | - | - | Streptococcus |
|  |  |  |  | - | - | - | - | -0.52 | -0.49 | Dolosigranulum |
|  |  |  | - | - | - | - | - | - | - | Moraxella |
|  |  | - | - | - | - | - | - | - | - | Propionibacterium |
|  | - | - | - | 0.69 | - | - | - | - | - | Corynebacterium |
| - | - | 0.52 | - | - | - | - | - | - | - | Staphylococcus |
| Staphylococcus | Corynebacterium | Propionibacterium | Moraxella | Dolosigranulum | Streptococcus | Peptoniphilus | Haemophilus | Streptophyta | Anaerococcus |  |
