# Supplementary material for: The administration of intranasal live attenuated influenza vaccine induces changes in the nasal microbiota and nasal epithelium gene expression profiles
Source: Microbiome. 2015 Dec 15;3:74. doi: 10.1186/s40168-015-0133-2 (PMC4678663; doi:10.1186/s40168-015-0133-2)
Supplement: Additional file 6: Table S2. — Significant correlations among the most abundant genera with control, LAIV, and FL samples. Similarly to Table S1, Pearson correlation coefficients (ρ) were calculated between the ALR transformed abundances for genera identified with the V1–V3 regions. Only genera with p values <0.05 (uncorrected for multiple testing) were provided below. The control, LAIV, and flu-like cohort consisted in total of 107 samples. In support to the results shown in Table S1, the positive correlation between Propionibacterium and Staphylococcus and between Dolosigranulum and Corynebacterium seem to be reinforced with the addition of the FL cohort. [file 40168_2015_133_MOESM6_ESM.docx]

Table S2. Significant correlations among the most abundant genera with Control, LAIV and FL Samples. Similarly to Table S1, Pearson correlation coefficients (ρ) were calculated between the ALR transformed abundances for genera identified with the V1V3 regions. Only genera with p-values < 0.05 (uncorrected for multiple testing) were provided below. The control, LAIV, and flu-like cohort consisted in total of 107 samples. In support to the results shown in Table S1, the positive correlation between Propionibacterium and Staphylococcus, and between Dolosigranulum and Corynebacterium seem to be reinforced with the addition of the FL cohort.

|  |  |  |  |  |  |  |  |  | - | Anaerococcus |
| --- | --- | --- | --- | --- | --- | --- | --- | --- | --- | --- |
|  |  |  |  |  |  |  |  | - | 0.33 | Streptophyta |
|  |  |  |  |  |  |  | - | - | - | Haemophilus |
|  |  |  |  |  |  | - | - | - | 0.62 | Peptoniphilus |
|  |  |  |  |  | - | - | 0.22 | - | - | Streptococcus |
|  |  |  |  | - | - | - | - | -0.21 | - | Dolosigranulum |
|  |  |  | - | 0.22 | - | - | - | - | - | Moraxella |
|  |  | - | - | - | -0.20 | 0.51 | - | 0.28 | 0.56 | Propionibacterium |
|  | - | 0.39 | 0.24 | 0.56 | - | 0.30 | - | - | 0.35 | Corynebacterium |
| - | 0.19 | 0.55 | - | - | -0.19 | 0.40 | - | 0.38 | 0.46 | Staphylococcus |
| Staphylococcus | Corynebacterium | Propionibacterium | Moraxella | Dolosigranulum | Streptococcus | Peptoniphilus | Haemophilus | Streptophyta | Anaerococcus |  |
