# Supplementary material for: The administration of intranasal live attenuated influenza vaccine induces changes in the nasal microbiota and nasal epithelium gene expression profiles
Source: Microbiome. 2015 Dec 15;3:74. doi: 10.1186/s40168-015-0133-2 (PMC4678663; doi:10.1186/s40168-015-0133-2)
Supplement: Additional file 7: Table S3. — Hypergeometric testing for the enrichment of GO/BP terms in the LAIV group. [file 40168_2015_133_MOESM7_ESM.docx]

**Table S3: Hypergeometric testing for the enrichment of GO:BP terms in the LAIV group**

|  | **GO:BP ID** | **P-value** | **Odds Ratio** | **Exp Count** | **Count** | **Size** | **Term** |
| --- | --- | --- | --- | --- | --- | --- | --- |
| **1** | GO:0006952 | 5.09E-08 | 7.89 | 3.50 | 16 | 765 | defense response |
| **2** | GO:0019884 | 4.51E-06 | 17.01 | 0.45 | 6 | 98 | antigen processing and presentation of exogenous antigen |
| **3** | GO:0002449 | 7.52E-06 | 15.47 | 0.49 | 6 | 107 | lymphocyte mediated immunity |
| **4** | GO:0048002 | 7.94E-06 | 15.32 | 0.49 | 6 | 108 | antigen processing and presentation of peptide antigen |
| **5** | GO:0002526 | 7.94E-06 | 22.19 | 0.28 | 5 | 62 | acute inflammatory response |
| **6** | GO:0002253 | 8.99E-06 | 9.54 | 1.09 | 8 | 239 | activation of immune response |
| **7** | GO:0002682 | 1.18E-05 | 5.99 | 2.88 | 12 | 629 | regulation of immune system process |
| **8** | GO:0002252 | 1.40E-05 | 7.76 | 1.54 | 9 | 337 | immune effector process |
| **9** | GO:0072376 | 1.54E-05 | 32.73 | 0.16 | 4 | 34 | protein activation cascade |
| **10** | GO:0030449 | 5.70E-05 | 51.01 | 0.08 | 3 | 17 | regulation of complement activation |
| **11** | GO:0006958 | 8.08E-05 | 44.63 | 0.09 | 3 | 19 | complement activation, classical pathway |
| **12** | GO:0060333 | 9.14E-05 | 19.99 | 0.24 | 4 | 53 | interferon-gamma-mediated signaling pathway |
| **13** | GO:0016064 | 0.0001 | 18.47 | 0.26 | 4 | 57 | immunoglobulin mediated immune response |
| **14** | GO:0019886 | 0.0001 | 17.79 | 0.27 | 4 | 59 | antigen processing and presentation of exogenous peptide antigen via MHC class II |
| **15** | GO:0002504 | 0.0002 | 17.16 | 0.28 | 4 | 61 | antigen processing and presentation of peptide or polysaccharide antigen via MHC class II |
| **16** | GO:0006959 | 0.0002 | 16.30 | 0.29 | 4 | 64 | humoral immune response |
| **17** | GO:0002824 | 0.0004 | 24.58 | 0.15 | 3 | 32 | positive regulation of adaptive immune response based on somatic recombination of immune receptors built from immunoglobulin superfamily domains |
| **18** | GO:0034341 | 0.0004 | 13.01 | 0.36 | 4 | 79 | response to interferon-gamma |
| **19** | GO:0002460 | 0.0004 | 13.00 | 0.36 | 4 | 87 | adaptive immune response based on somatic recombination of immune receptors built from immunoglobulin superfamily domains |
| **20** | GO:0006957 | 0.0006 | 76.96 | 0.04 | 2 | 8 | complement activation, alternative pathway |
| **21** | GO:0050778 | 0.0006 | 21.10 | 0.17 | 3 | 47 | positive regulation of immune response |
| **22** | GO:0071345 | 0.0009 | 6.15 | 1.17 | 6 | 255 | cellular response to cytokine stimulus |

Gene selection thresholds are p value < 0.01 and log_2_FC > 0.7.
