# Supplementary material for: The administration of intranasal live attenuated influenza vaccine induces changes in the nasal microbiota and nasal epithelium gene expression profiles
Source: Microbiome. 2015 Dec 15;3:74. doi: 10.1186/s40168-015-0133-2 (PMC4678663; doi:10.1186/s40168-015-0133-2)
Supplement: Additional file 8: Table S4. — Hypergeometric testing for the enrichment of GO/BP terms in the control group. [file 40168_2015_133_MOESM8_ESM.docx]

**Table S4: Hypergeometric testing for the enrichment of GO:BP terms in the control group**

|  | **GO:BP ID** | **P-value** | **Odds Ratio** | **Exp Count** | **Count** | **Size** | **Term** |
| --- | --- | --- | --- | --- | --- | --- | --- |
| **1** | GO:0001539 | 2.766E-06 | 2.909E+01 | 2.346E-01 | 5 | 22 | ciliary or bacterial-type flagellar motility |
| **2** | GO:0060271 | 7.805E-06 | 9.180E+00 | 1.034E+00 | 8 | 97 | cilium morphogenesis |
| **3** | GO:0044782 | 2.029E-05 | 9.677E+00 | 8.529E-01 | 7 | 80 | cilium organization |
| **4** | GO:0003341 | 6.236E-05 | 2.296E+01 | 2.239E-01 | 4 | 21 | cilium movement |
| **5** | GO:0035083 | 6.283E-05 | 5.786E+01 | 8.529E-02 | 3 | 8 | cilium axoneme assembly |
| **6** | GO:0007018 | 1.689E-04 | 8.295E+00 | 8.318E-01 | 6 | 82 | microtubule-based movement |
| **7** | GO:0030031 | 2.163E-04 | 5.551E+00 | 1.642E+00 | 8 | 154 | cell projection assembly |
| **8** | GO:0048609 | 7.051E-04 | 3.549E+00 | 3.529E+00 | 11 | 331 | multicellular organismal reproductive process |

Gene selection thresholds are p value < 0.01 and log_2_FC > 0.7.
