# Supplementary material for: The administration of intranasal live attenuated influenza vaccine induces changes in the nasal microbiota and nasal epithelium gene expression profiles
Source: Microbiome. 2015 Dec 15;3:74. doi: 10.1186/s40168-015-0133-2 (PMC4678663; doi:10.1186/s40168-015-0133-2)
Supplement: Additional file 9: Table S5. — Type I and type II Interferon-stimulated gene list. [file 40168_2015_133_MOESM9_ESM.docx]

**Table S5. Type I and Type II Interferon stimulated gene list**

| Gene Name | Type | Ensembl ID | Probe ID |
| --- | --- | --- | --- |
| ACAP2 | I | ENSG00000114331 | 212477_at |
| ACAP2 | II | ENSG00000114331 | 212477_at |
| ADAM22 | II | ENSG00000008277 | 213411_at |
| ADAMTS6 | I | ENSG00000049192 | 220866_at |
| AHNAK | II | ENSG00000124942 | 211986_at |
| AHNAK | II | ENSG00000124942 | 220016_at |
| ANGPT1 | I | ENSG00000154188 | 205609_at |
| APBB1IP | I | ENSG00000077420 | 219994_at |
| APOL6 | II | ENSG00000221963 | 219716_at |
| ARID5B | II | ENSG00000150347 | 212614_at |
| ATF3 | II | ENSG00000162772 | 202672_s_at |
| BTRC | II | ENSG00000166167 | 222374_at |
| C19orf66 | I | ENSG00000130813 | 53720_at |
| C19orf66 | I | ENSG00000130813 | 218429_s_at |
| C19orf66 | II | ENSG00000130813 | 218429_s_at |
| C1S | II | ENSG00000182326 | 208747_s_at |
| C1S | I | ENSG00000182326 | 208747_s_at |
| CADPS | I | ENSG00000163618 | 204814_at |
| CALCOCO2 | II | ENSG00000136436 | 210817_s_at |
| CCND2 | II | ENSG00000118971 | 200952_s_at |
| CD6 | I | ENSG00000013725 | 213958_at |
| CD74 | II | ENSG00000019582 | 209619_at |
| CD8B | I | ENSG00000172116 | 207979_s_at |
| CNTN1 | II | ENSG00000018236 | 211203_s_at |
| COL1A1 | I | ENSG00000108821 | 202310_s_at |
| CPE | I | ENSG00000109472 | 201116_s_at |
| CTNND2 | I | ENSG00000169862 | 209618_at |
| CXCL2 | II | ENSG00000081041 | 209774_x_at |
| DOCK4 | II | ENSG00000128512 | 205003_at |
| DPYD | II | ENSG00000188641 | 204646_at |
| ERAP2 | II | ENSG00000164308 | 219759_at |
| ERCC8 | I | ENSG00000049167 | 220716_at |
| FGF1 | II | ENSG00000113578 | 205117_at |
| FGFR4 | II | ENSG00000160867 | 211237_s_at |
| GAB1 | I | ENSG00000109458 | 207112_s_at |
| GRK4 | II | ENSG00000125388 | 208365_s_at |
| GUK1 | II | ENSG00000143774 | 200075_s_at |
| HERC6 | I | ENSG00000138642 | 219352_at |
| HFE | II | ENSG00000010704 | 211331_x_at |
| HLA-E | II | ENSG00000204592 | 200904_at |
| HLA-E | II | ENSG00000204592 | 200905_x_at |
| HLA-E | II | ENSG00000204592 | 217456_x_at |
| HLA-E | I | ENSG00000204592 | 200905_x_at |
| HNMT | I | ENSG00000150540 | 204111_at |
| HNRPDL | I | ENSG00000152795 | 214379_at |
| HSPA6 | II | ENSG00000173110 | 213418_at |
| IFIH1 | II | ENSG00000115267 | 219209_at |
| IFIH1 | I | ENSG00000115267 | 219209_at |
| IGFBP5 | I | ENSG00000115461 | 203425_s_at |
| JAK2 | II | ENSG00000096968 | 205842_s_at |
| JUP | II | ENSG00000173801 | 201015_s_at |
| LGALS3BP | II | ENSG00000108679 | 200923_at |
| LGALS3BP | I | ENSG00000108679 | 200923_at |
| MAP3K19 | II | ENSG00000176601 | 221180_at |
| MCAM | II | ENSG00000076706 | 210869_s_at |
| MCL1 | II | ENSG00000143384 | 200796_s_at |
| MCL1 | II | ENSG00000143384 | 200797_s_at |
| MCL1 | II | ENSG00000143384 | 200798_x_at |
| MUC4 | I | ENSG00000145113 | 204895_x_at |
| NAMPT | II | ENSG00000105835 | 217738_at |
| NAMPT | II | ENSG00000105835 | 217739_s_at |
| NMNAT2 | II | ENSG00000157064 | 209755_at |
| NT5DC2 | II | ENSG00000168268 | 218051_s_at |
| NUCB1 | II | ENSG00000104805 | 200646_s_at |
| NUCB1 | II | ENSG00000104805 | 200649_at |
| OR2H1 | I | ENSG00000204688 | 216817_s_at |
| PALLD | II | ENSG00000129116 | 200906_s_at |
| PCSK6 | II | ENSG00000140479 | 211263_s_at |
| PDE4D | II | ENSG00000113448 | 211840_s_at |
| PSME1 | II | ENSG00000092010 | 200814_at |
| RALGPS1 | II | ENSG00000136828 | 210552_s_at |
| SAMHD1 | I | ENSG00000101347 | 204502_at |
| SAMHD1 | II | ENSG00000101347 | 204502_at |
| SERPING1 | II | ENSG00000149131 | 200986_at |
| SFXN3 | II | ENSG00000107819 | 217226_s_at |
| SLC9A7 | II | ENSG00000065923 | 214860_at |
| SLC9A7 | II | ENSG00000271342 | 214860_at |
| SP140L | I | ENSG00000185404 | 214791_at |
| SP140L | II | ENSG00000185404 | 214791_at |
| SRPK2 | I | ENSG00000135250 | 217517_x_at |
| ST8SIA4 | II | ENSG00000113532 | 206925_at |
| STAT1 | II | ENSG00000115415 | 200887_s_at |
| STAT1 | I | ENSG00000115415 | 209969_s_at |
| STAT1 | I | ENSG00000115415 | 200887_s_at |
| STAT1 | II | ENSG00000115415 | 209969_s_at |
| SYNJ2 | II | ENSG00000078269 | 210612_s_at |
| TAPBP | I | ENSG00000112493 | 208829_at |
| TAPBP | II | ENSG00000231925 | 208829_at |
| TAPBP | I | ENSG00000231925 | 208829_at |
| TAPBP | II | ENSG00000112493 | 208829_at |
| TGM2 | II | ENSG00000198959 | 201042_at |
| TGM2 | II | ENSG00000198959 | 211573_x_at |
| TGM2 | II | ENSG00000198959 | 211003_x_at |
| THRA | II | ENSG00000126351 | 214883_at |
| TLL1 | I | ENSG00000038295 | 206415_at |
| TTLL3 | II | ENSG00000214021 | 210129_s_at |
| UBA7 | II | ENSG00000182179 | 1294_at |
| UBA7 | II | ENSG00000182179 | 203281_s_at |
| VASH2 | II | ENSG00000143494 | 219740_at |
| WARS | II | ENSG00000140105 | 200628_s_at |
| WARS | II | ENSG00000140105 | 200629_at |
| ZNF230 | I | ENSG00000159882 | 205791_x_at |
| ZNF277 | II | ENSG00000198839 | 218645_at |
| ADAM11 | II | ENSG00000073670 | 207880_at |
| ADAM20 | II | ENSG00000134007 | 207422_at |
| ADAR | II | ENSG00000160710 | 201786_s_at |
| ADAR | I | ENSG00000160710 | 201786_s_at |
| AGAP2 | I | ENSG00000135439 | 215080_s_at |
| AGTPBP1 | I | ENSG00000135049 | 204499_at |
| AGXT | I | ENSG00000172482 | 210326_at |
| ALPP | I | ENSG00000163283 | 211619_s_at |
| ALPP | II | ENSG00000163283 | 211619_s_at |
| AOX1 | II | ENSG00000138356 | 205083_at |
| AP1G2 | II | ENSG00000213983 | 214341_at |
| AQP3 | II | ENSG00000165272 | 39248_at |
| AQP3 | II | ENSG00000165272 | 203747_at |
| ARL4C | II | ENSG00000188042 | 213759_at |
| ART4 | II | ENSG00000111339 | 207220_at |
| ATP2C2 | II | ENSG00000064270 | 206043_s_at |
| ATP4A | I | ENSG00000105675 | 207139_at |
| ATP4A | II | ENSG00000105675 | 207139_at |
| B2M | I | ENSG00000166710 | 216231_s_at |
| B2M | II | ENSG00000166710 | 201891_s_at |
| B2M | II | ENSG00000166710 | 216231_s_at |
| B2M | I | ENSG00000166710 | 201891_s_at |
| BAAT | II | ENSG00000136881 | 206913_at |
| BACH1 | II | ENSG00000156273 | 204194_at |
| BMP2 | I | ENSG00000125845 | 205289_at |
| BMP6 | II | ENSG00000153162 | 215042_at |
| BST2 | II | ENSG00000130303 | 201641_at |
| BST2 | I | ENSG00000130303 | 201641_at |
| BTN3A3 | II | ENSG00000111801 | 204820_s_at |
| BTN3A3 | II | ENSG00000111801 | 38241_at |
| BTN3A3 | II | ENSG00000111801 | 204821_at |
| CALB1 | I | ENSG00000104327 | 205625_s_at |
| CASP1 | II | ENSG00000137752 | 211366_x_at |
| CASP1 | II | ENSG00000137752 | 211367_s_at |
| CASP1 | II | ENSG00000137752 | 211368_s_at |
| CASP1 | II | ENSG00000137752 | 209970_x_at |
| CASP7 | II | ENSG00000165806 | 207181_s_at |
| CAT | II | ENSG00000121691 | 215573_at |
| CD22 | II | ENSG00000012124 | 220674_at |
| CD2AP | II | ENSG00000198087 | 203593_at |
| CD38 | II | ENSG00000004468 | 205692_s_at |
| CD40 | II | ENSG00000101017 | 205153_s_at |
| CD40 | II | ENSG00000101017 | 215346_at |
| CD86 | I | ENSG00000114013 | 205686_s_at |
| CDC14A | II | ENSG00000079335 | 210742_at |
| CEACAM1 | II | ENSG00000079385 | 211883_x_at |
| CEACAM1 | II | ENSG00000079385 | 209498_at |
| CFB | I | ENSG00000244255 | 202357_s_at |
| CFB | I | ENSG00000239754 | 202357_s_at |
| CFB | II | ENSG00000244255 | 202357_s_at |
| CFB | II | ENSG00000239754 | 202357_s_at |
| CIB2 | II | ENSG00000136425 | 214065_s_at |
| CLDN3 | II | ENSG00000165215 | 203954_x_at |
| CLTB | II | ENSG00000175416 | 221996_s_at |
| CPM | II | ENSG00000135678 | 206100_at |
| CRH | I | ENSG00000147571 | 205629_s_at |
| CRH | I | ENSG00000147571 | 205630_at |
| CST2 | II | ENSG00000170369 | 208555_x_at |
| CTDSPL | II | ENSG00000144677 | 201904_s_at |
| CTSL1 | II | ENSG00000135047 | 202087_s_at |
| CTSS | II | ENSG00000163131 | 202901_x_at |
| CTSS | II | ENSG00000163131 | 202902_s_at |
| CUL3 | I | ENSG00000036257 | 201372_s_at |
| CUL3 | II | ENSG00000036257 | 201372_s_at |
| CXCL3 | II | ENSG00000163734 | 207850_at |
| CXCL5 | II | ENSG00000163735 | 214974_x_at |
| CXCL5 | II | ENSG00000163735 | 215101_s_at |
| CXCL5 | II | ENSG00000163735 | 207852_at |
| CYP27A1 | II | ENSG00000135929 | 203979_at |
| CYP3A43 | II | ENSG00000021461 | 211442_x_at |
| CYP3A5 | II | ENSG00000106258 | 214235_at |
| CYTH1 | II | ENSG00000108669 | 202879_s_at |
| DIO2 | I | ENSG00000211448 | 203699_s_at |
| DKK3 | II | ENSG00000050165 | 221126_at |
| DNAJC4 | I | ENSG00000110011 | 206782_s_at |
| DPT | I | ENSG00000143196 | 213068_at |
| EFS | II | ENSG00000100842 | 210880_s_at |
| EGR2 | I | ENSG00000122877 | 205249_at |
| EIF2AK2 | I | ENSG00000055332 | 213294_at |
| EIF2AK2 | I | ENSG00000055332 | 204211_x_at |
| ELF4 | I | ENSG00000102034 | 203490_at |
| EML2 | II | ENSG00000125746 | 204399_s_at |
| EMR1 | I | ENSG00000174837 | 207111_at |
| EMR1 | II | ENSG00000174837 | 207111_at |
| FANCA | II | ENSG00000187741 | 203806_s_at |
| FAS | II | ENSG00000026103 | 216252_x_at |
| FAS | II | ENSG00000026103 | 215719_x_at |
| FBLN1 | II | ENSG00000077942 | 202994_s_at |
| FGF9 | II | ENSG00000102678 | 206404_at |
| FGFR2 | II | ENSG00000066468 | 211400_at |
| FLT3LG | II | ENSG00000090554 | 206980_s_at |
| FOSL2 | II | ENSG00000075426 | 218880_at |
| FOXN3 | II | ENSG00000053254 | 218031_s_at |
| FUT9 | I | ENSG00000172461 | 216185_at |
| FUT9 | II | ENSG00000172461 | 216185_at |
| GABBR1 | II | ENSG00000204681 | 205890_s_at |
| GAL3ST1 | II | ENSG00000128242 | 205670_at |
| GAP43 | I | ENSG00000172020 | 216963_s_at |
| GBP1 | II | ENSG00000117228 | 202269_x_at |
| GBP1 | II | ENSG00000117228 | 202270_at |
| GBP1 | I | ENSG00000117228 | 202269_x_at |
| GBP1 | I | ENSG00000117228 | 202270_at |
| GBP2 | II | ENSG00000162645 | 202748_at |
| GCM1 | I | ENSG00000137270 | 206269_at |
| GH1 | I | ENSG00000259384 | 211151_x_at |
| GH1 | II | ENSG00000259384 | 211151_x_at |
| GH2 | I | ENSG00000136487 | 211151_x_at |
| GH2 | II | ENSG00000136487 | 211151_x_at |
| GLS | I | ENSG00000115419 | 203157_s_at |
| GLS | II | ENSG00000115419 | 203157_s_at |
| GLS | II | ENSG00000115419 | 203159_at |
| GNL3L | I | ENSG00000130119 | 217632_at |
| GPRC5B | II | ENSG00000167191 | 203632_s_at |
| HLA-B | II | ENSG00000206450 | 211911_x_at |
| HLA-B | II | ENSG00000234745 | 208729_x_at |
| HLA-B | II | ENSG00000232126 | 211911_x_at |
| HLA-B | I | ENSG00000206450 | 209140_x_at |
| HLA-B | I | ENSG00000234745 | 209140_x_at |
| HLA-B | I | ENSG00000206450 | 211911_x_at |
| HLA-B | II | ENSG00000206450 | 209140_x_at |
| HLA-B | I | ENSG00000224608 | 208729_x_at |
| HLA-B | I | ENSG00000232126 | 211911_x_at |
| HLA-B | II | ENSG00000234745 | 209140_x_at |
| HLA-B | I | ENSG00000234745 | 208729_x_at |
| HLA-B | II | ENSG00000224608 | 208729_x_at |
| HLA-DOA | I | ENSG00000204252 | 211142_x_at |
| HLA-F | I | ENSG00000137403 | 204806_x_at |
| HLA-F | II | ENSG00000204642 | 221875_x_at |
| HLA-F | I | ENSG00000204642 | 221875_x_at |
| HLA-F | II | ENSG00000137403 | 221875_x_at |
| HLA-F | II | ENSG00000204642 | 204806_x_at |
| HLA-F | I | ENSG00000137403 | 221875_x_at |
| HLA-F | II | ENSG00000137403 | 204806_x_at |
| HLA-F | I | ENSG00000204642 | 204806_x_at |
| HPGDS | II | ENSG00000163106 | 206726_at |
| HSD3B2 | I | ENSG00000203859 | 206294_at |
| HTR1D | II | ENSG00000179546 | 207368_at |
| ICA1 | I | ENSG00000003147 | 211740_at |
| ICAM1 | II | ENSG00000090339 | 215485_s_at |
| ICAM1 | II | ENSG00000090339 | 202637_s_at |
| ICAM1 | II | ENSG00000090339 | 202638_s_at |
| IFI16 | I | ENSG00000163565 | 208966_x_at |
| IFI16 | I | ENSG00000163565 | 206332_s_at |
| IFI16 | II | ENSG00000163565 | 206332_s_at |
| IFI16 | II | ENSG00000163565 | 208965_s_at |
| IFI16 | II | ENSG00000163565 | 208966_x_at |
| IFI27 | I | ENSG00000165949 | 202411_at |
| IFI27 | II | ENSG00000165949 | 202411_at |
| IFI44L | I | ENSG00000137959 | 204439_at |
| IFI44L | II | ENSG00000137959 | 204439_at |
| IFI6 | II | ENSG00000126709 | 204415_at |
| IFI6 | I | ENSG00000126709 | 204415_at |
| IFIT1 | I | ENSG00000185745 | 203153_at |
| IFIT1 | II | ENSG00000185745 | 203153_at |
| IFIT3 | I | ENSG00000119917 | 204747_at |
| IFIT3 | II | ENSG00000119917 | 204747_at |
| IFIT5 | I | ENSG00000152778 | 203595_s_at |
| IFIT5 | I | ENSG00000152778 | 203596_s_at |
| IFIT5 | II | ENSG00000152778 | 203595_s_at |
| IFIT5 | II | ENSG00000152778 | 203596_s_at |
| IFITM2 | II | ENSG00000185201 | 201601_x_at |
| IFITM2 | II | ENSG00000185201 | 201315_x_at |
| IFITM2 | I | ENSG00000185201 | 201601_x_at |
| IFITM2 | I | ENSG00000185201 | 214022_s_at |
| IFITM2 | II | ENSG00000185201 | 214022_s_at |
| IFITM2 | I | ENSG00000185201 | 201315_x_at |
| IL15 | II | ENSG00000164136 | 217371_s_at |
| IL15RA | II | ENSG00000134470 | 207375_s_at |
| IL1R2 | I | ENSG00000115590 | 205403_at |
| IL32 | II | ENSG00000008517 | 203828_s_at |
| IL7 | II | ENSG00000104432 | 206693_at |
| IRF1 | II | ENSG00000125347 | 202531_at |
| IRF7 | I | ENSG00000185507 | 208436_s_at |
| IRF7 | II | ENSG00000185507 | 208436_s_at |
| IRF8 | II | ENSG00000140968 | 204057_at |
| IRF9 | I | ENSG00000213928 | 203882_at |
| IRF9 | II | ENSG00000213928 | 203882_at |
| ISG15 | II | ENSG00000187608 | 205483_s_at |
| ISG15 | I | ENSG00000187608 | 205483_s_at |
| ISG20 | II | ENSG00000172183 | 204698_at |
| ISG20 | II | ENSG00000172183 | 33304_at |
| ISG20 | I | ENSG00000172183 | 204698_at |
| ISG20 | I | ENSG00000172183 | 33304_at |
| ITGB3 | I | ENSG00000259207 | 211579_at |
| ITGB3 | II | ENSG00000259207 | 204626_s_at |
| IVD | I | ENSG00000128928 | 203682_s_at |
| JUNB | II | ENSG00000171223 | 201473_at |
| KALRN | I | ENSG00000160145 | 205635_at |
| KALRN | II | ENSG00000160145 | 205635_at |
| KCNAB1 | II | ENSG00000169282 | 208213_s_at |
| KCNJ2 | II | ENSG00000123700 | 206765_at |
| KDELR3 | II | ENSG00000100196 | 207264_at |
| KRT15 | II | ENSG00000171346 | 204734_at |
| KRT19 | II | ENSG00000171345 | 201650_at |
| KRT5 | I | ENSG00000186081 | 201820_at |
| L3MBTL1 | II | ENSG00000185513 | 206823_at |
| LAMP3 | II | ENSG00000078081 | 205569_at |
| LAMP3 | I | ENSG00000078081 | 205569_at |
| LEPREL4 | II | ENSG00000141696 | 204078_at |
| LILRB1 | II | ENSG00000104972 | 211336_x_at |
| LIPF | II | ENSG00000182333 | 206334_at |
| LOX | II | ENSG00000113083 | 215446_s_at |
| LRRC1 | I | ENSG00000137269 | 207790_at |
| LSR | II | ENSG00000105699 | 208190_s_at |
| LTBP1 | II | ENSG00000049323 | 202728_s_at |
| LY6E | I | ENSG00000160932 | 202145_at |
| LY86 | I | ENSG00000112799 | 205859_at |
| MAFF | II | ENSG00000185022 | 36711_at |
| MAGEC1 | II | ENSG00000155495 | 206609_at |
| MAPT | II | ENSG00000186868 | 203928_x_at |
| MDC1 | II | ENSG00000137337 | 203061_s_at |
| MEP1B | I | ENSG00000141434 | 207251_at |
| MMP24 | II | ENSG00000125966 | 213171_s_at |
| MPPE1 | II | ENSG00000154889 | 209858_x_at |
| MT1H | II | ENSG00000205358 | 211456_x_at |
| MTM1 | I | ENSG00000171100 | 36920_at |
| MTTP | II | ENSG00000138823 | 205675_at |
| MX1 | I | ENSG00000157601 | 202086_at |
| MX1 | II | ENSG00000157601 | 202086_at |
| MX2 | II | ENSG00000183486 | 204994_at |
| MX2 | I | ENSG00000183486 | 204994_at |
| NBN | II | ENSG00000104320 | 217299_s_at |
| NCOR2 | I | ENSG00000196498 | 208889_s_at |
| NF1 | I | ENSG00000196712 | 211095_at |
| NF1 | I | ENSG00000196712 | 211914_x_at |
| NFE2L3 | II | ENSG00000050344 | 204702_s_at |
| NMI | II | ENSG00000123609 | 203964_at |
| NMI | I | ENSG00000123609 | 203964_at |
| NNMT | II | ENSG00000166741 | 202238_s_at |
| NPC1 | II | ENSG00000141458 | 202679_at |
| OAS1 | II | ENSG00000089127 | 205552_s_at |
| OAS1 | I | ENSG00000089127 | 202869_at |
| OAS1 | II | ENSG00000089127 | 202869_at |
| OAS1 | I | ENSG00000089127 | 205552_s_at |
| OAS2 | II | ENSG00000111335 | 206553_at |
| OAS2 | II | ENSG00000111335 | 204972_at |
| OAS2 | I | ENSG00000111335 | 204972_at |
| OAS2 | I | ENSG00000111335 | 206553_at |
| OASL | I | ENSG00000135114 | 210797_s_at |
| OASL | II | ENSG00000135114 | 210797_s_at |
| OASL | II | ENSG00000135114 | 205660_at |
| OASL | I | ENSG00000135114 | 205660_at |
| OCM2 | II | ENSG00000135175 | 207944_at |
| OGFR | I | ENSG00000060491 | 210443_x_at |
| OGFR | II | ENSG00000060491 | 211512_s_at |
| OGFR | II | ENSG00000060491 | 210443_x_at |
| OGFR | II | ENSG00000060491 | 211513_s_at |
| OGFR | II | ENSG00000060491 | 202841_x_at |
| OPRD1 | I | ENSG00000116329 | 207792_at |
| OPTN | II | ENSG00000123240 | 202074_s_at |
| PAX2 | II | ENSG00000075891 | 206228_at |
| PDPK1 | I | ENSG00000140992 | 221244_s_at |
| PDX1 | I | ENSG00000139515 | 210938_at |
| PLAT | II | ENSG00000104368 | 201860_s_at |
| PLCH2 | I | ENSG00000149527 | 206080_at |
| PLSCR1 | II | ENSG00000188313 | 202446_s_at |
| PLSCR1 | I | ENSG00000188313 | 202430_s_at |
| PLSCR1 | I | ENSG00000188313 | 202446_s_at |
| PLSCR1 | II | ENSG00000188313 | 202430_s_at |
| PMAIP1 | II | ENSG00000141682 | 204286_s_at |
| PMAIP1 | II | ENSG00000141682 | 204285_s_at |
| PMCH | II | ENSG00000183395 | 206942_s_at |
| PML | II | ENSG00000140464 | 206503_x_at |
| PML | II | ENSG00000140464 | 210362_x_at |
| PML | II | ENSG00000140464 | 211012_s_at |
| PML | I | ENSG00000140464 | 206503_x_at |
| PML | II | ENSG00000140464 | 211013_x_at |
| PML | I | ENSG00000140464 | 211013_x_at |
| POLG | II | ENSG00000140521 | 217636_at |
| POU6F1 | II | ENSG00000184271 | 205878_at |
| PPL | II | ENSG00000118898 | 203407_at |
| PPP1R3C | II | ENSG00000119938 | 204284_at |
| PRMT8 | II | ENSG00000111218 | 207772_s_at |
| PRSS23 | II | ENSG00000150687 | 202458_at |
| PSMB10 | II | ENSG00000205220 | 202659_at |
| PSMB9 | I | ENSG00000239836 | 204279_at |
| PSMB9 | II | ENSG00000239836 | 204279_at |
| PSMB9 | I | ENSG00000240065 | 204279_at |
| PSMB9 | II | ENSG00000240065 | 204279_at |
| PSME2 | II | ENSG00000100911 | 201762_s_at |
| PSME2 | I | ENSG00000100911 | 201762_s_at |
| PTGER3 | I | ENSG00000050628 | 208169_s_at |
| PTGER3 | II | ENSG00000050628 | 210831_s_at |
| RAB3B | II | ENSG00000169213 | 205925_s_at |
| RAB40B | II | ENSG00000141542 | 204547_at |
| RAE1 | II | ENSG00000101146 | 211319_at |
| RAP1GAP | II | ENSG00000076864 | 203911_at |
| RARRES1 | II | ENSG00000118849 | 221872_at |
| RARRES1 | II | ENSG00000118849 | 206392_s_at |
| RARRES3 | II | ENSG00000133321 | 204070_at |
| RARRES3 | I | ENSG00000133321 | 204070_at |
| REEP1 | II | ENSG00000068615 | 204364_s_at |
| REEP1 | I | ENSG00000068615 | 204364_s_at |
| RFPL2 | I | ENSG00000128253 | 207227_x_at |
| RFPL2 | II | ENSG00000128253 | 207227_x_at |
| RNF125 | I | ENSG00000101695 | 207735_at |
| RNF24 | II | ENSG00000101236 | 204669_s_at |
| ROR1 | I | ENSG00000185483 | 211057_at |
| ROR2 | I | ENSG00000169071 | 205578_at |
| RREB1 | II | ENSG00000124782 | 216648_s_at |
| SCNN1A | II | ENSG00000111319 | 217264_s_at |
| SCNN1G | II | ENSG00000166828 | 207295_at |
| SCYL3 | II | ENSG00000000457 | 205607_s_at |
| SEMA3F | II | ENSG00000001617 | 206832_s_at |
| SEZ6L | I | ENSG00000100095 | 213609_s_at |
| SLC14A2 | II | ENSG00000132874 | 208409_at |
| SLC15A2 | I | ENSG00000163406 | 205317_s_at |
| SLC2A3 | II | ENSG00000059804 | 202497_x_at |
| SLC35A3 | II | ENSG00000117620 | 209865_at |
| SLC7A8 | I | ENSG00000092068 | 216604_s_at |
| SLCO1A2 | II | ENSG00000084453 | 211481_at |
| SLIT3 | II | ENSG00000184347 | 203813_s_at |
| SNX1 | II | ENSG00000028528 | 216357_at |
| SOCS3 | II | ENSG00000184557 | 206359_at |
| SORL1 | II | ENSG00000137642 | 212560_at |
| SOX9 | II | ENSG00000125398 | 202935_s_at |
| SP100 | II | ENSG00000067066 | 202863_at |
| SP100 | I | ENSG00000067066 | 202864_s_at |
| SP100 | II | ENSG00000067066 | 210218_s_at |
| SP100 | II | ENSG00000067066 | 202864_s_at |
| SP100 | II | ENSG00000067066 | 210985_s_at |
| SP100 | I | ENSG00000067066 | 210218_s_at |
| SP100 | I | ENSG00000067066 | 202863_at |
| SP110 | II | ENSG00000135899 | 209761_s_at |
| SP110 | II | ENSG00000135899 | 209762_x_at |
| SP110 | II | ENSG00000135899 | 208392_x_at |
| SP110 | I | ENSG00000135899 | 208392_x_at |
| SP110 | II | ENSG00000135899 | 208012_x_at |
| SP110 | I | ENSG00000135899 | 209761_s_at |
| SP110 | I | ENSG00000135899 | 209762_x_at |
| SP110 | I | ENSG00000135899 | 208012_x_at |
| SRY | II | ENSG00000184895 | 207893_at |
| ST3GAL2 | I | ENSG00000157350 | 217650_x_at |
| STAT2 | II | ENSG00000170581 | 205170_at |
| TAP1 | II | ENSG00000168394 | 202307_s_at |
| TAP1 | I | ENSG00000168394 | 202307_s_at |
| TAP2 | II | ENSG00000204267 | 204770_at |
| TAP2 | II | ENSG00000204267 | 204769_s_at |
| TBX1 | I | ENSG00000184058 | 211274_at |
| TCL1B | I | ENSG00000213231 | 206413_s_at |
| TIMP4 | II | ENSG00000157150 | 206243_at |
| TLR3 | I | ENSG00000164342 | 206271_at |
| TLR3 | II | ENSG00000164342 | 206271_at |
| TMEM63A | I | ENSG00000196187 | 215583_at |
| TNFRSF1B | I | ENSG00000028137 | 203508_at |
| TOX | I | ENSG00000198846 | 204530_s_at |
| TPSAB1 | I | ENSG00000172236 | 216474_x_at |
| TRAFD1 | II | ENSG00000135148 | 35254_at |
| TRAFD1 | II | ENSG00000135148 | 202837_at |
| TREX1 | I | ENSG00000213689 | 205875_s_at |
| TREX1 | II | ENSG00000213689 | 205875_s_at |
| TRIM14 | I | ENSG00000106785 | 203148_s_at |
| TRIM14 | II | ENSG00000106785 | 203148_s_at |
| TRIM14 | I | ENSG00000106785 | 211044_at |
| TRIM21 | II | ENSG00000132109 | 204804_at |
| TRIM21 | I | ENSG00000132109 | 204804_at |
| TRIM31 | II | ENSG00000204616 | 215444_s_at |
| TRIM31 | II | ENSG00000137397 | 215444_s_at |
| TRIM31 | II | ENSG00000204616 | 208170_s_at |
| TRIM31 | II | ENSG00000204616 | 210159_s_at |
| TRIM31 | II | ENSG00000137397 | 208170_s_at |
| TRIM31 | II | ENSG00000137397 | 210159_s_at |
| TRIM38 | II | ENSG00000112343 | 203567_s_at |
| TRIM38 | II | ENSG00000112343 | 203568_s_at |
| TRIM38 | II | ENSG00000112343 | 203610_s_at |
| TYMP | II | ENSG00000025708 | 204858_s_at |
| TYMP | I | ENSG00000025708 | 204858_s_at |
| UBE2L6 | II | ENSG00000156587 | 201649_at |
| UBE2L6 | I | ENSG00000156587 | 201649_at |
| UBL3 | II | ENSG00000122042 | 201534_s_at |
| UGCG | I | ENSG00000148154 | 221765_at |
| UPK3B | II | ENSG00000243566 | 206658_at |
| UTY | I | ENSG00000183878 | 211149_at |
| XAF1 | II | ENSG00000132530 | 206133_at |
| XCL1 | II | ENSG00000143184 | 206366_x_at |
| ZFP36 | II | ENSG00000128016 | 201531_at |
| ZMYND10 | II | ENSG00000004838 | 205714_s_at |
| ZSCAN9 | I | ENSG00000137185 | 205181_at |
| ACSL5 | II | ENSG00000197142 | 218322_s_at |
| ACVR2B-AS1 | II | ENSG00000229589 | 215590_x_at |
| ADAMTS2 | II | ENSG00000087116 | 214535_s_at |
| ADAMTS7 | II | ENSG00000136378 | 220706_at |
| ADCY1 | II | ENSG00000164742 | 215340_at |
| ADH1B | II | ENSG00000196616 | 209612_s_at |
| ALPK3 | I | ENSG00000136383 | 214846_s_at |
| ALPPL2 | I | ENSG00000163286 | 211619_s_at |
| ALPPL2 | II | ENSG00000163286 | 211619_s_at |
| ANKRD2 | II | ENSG00000165887 | 221232_s_at |
| AP1M2 | II | ENSG00000129354 | 65517_at |
| AP4E1 | II | ENSG00000081014 | 220228_at |
| AP4E1 | II | ENSG00000081014 | 220229_s_at |
| AP4E1 | I | ENSG00000081014 | 220228_at |
| AP4E1 | I | ENSG00000081014 | 220229_s_at |
| AP5Z1 | II | ENSG00000242802 | 209912_s_at |
| APOL1 | I | ENSG00000100342 | 209546_s_at |
| APOL1 | II | ENSG00000100342 | 209546_s_at |
| APOL2 | II | ENSG00000128335 | 221013_s_at |
| APOL2 | II | ENSG00000128335 | 221653_x_at |
| APOL3 | II | ENSG00000128284 | 221087_s_at |
| ATG16L1 | II | ENSG00000085978 | 220521_s_at |
| ATP10D | II | ENSG00000145246 | 213238_at |
| BATF3 | II | ENSG00000123685 | 220358_at |
| BCMO1 | II | ENSG00000135697 | 220087_at |
| BMPR1B | I | ENSG00000138696 | 210523_at |
| BSPRY | II | ENSG00000119411 | 218792_s_at |
| BTN3A2 | II | ENSG00000186470 | 212613_at |
| BTN3A2 | II | ENSG00000186470 | 204820_s_at |
| BTN3A2 | II | ENSG00000186470 | 209846_s_at |
| C1R | II | ENSG00000159403 | 212067_s_at |
| C1R | I | ENSG00000159403 | 212067_s_at |
| C1RL | II | ENSG00000139178 | 218983_at |
| C3 | II | ENSG00000125730 | 217767_at |
| C3 | I | ENSG00000125730 | 217767_at |
| CASP4 | II | ENSG00000196954 | 209310_s_at |
| CCDC68 | II | ENSG00000166510 | 220180_at |
| CCL8 | I | ENSG00000108700 | 214038_at |
| CD47 | II | ENSG00000196776 | 213857_s_at |
| CDC42EP4 | II | ENSG00000179604 | 218063_s_at |
| CDK18 | II | ENSG00000117266 | 214797_s_at |
| CDK18 | I | ENSG00000117266 | 214797_s_at |
| CDKN1C | I | ENSG00000129757 | 213183_s_at |
| CDKN1C | II | ENSG00000129757 | 213183_s_at |
| CEP152 | I | ENSG00000103995 | 215882_at |
| CERK | II | ENSG00000100422 | 218421_at |
| CFH | II | ENSG00000000971 | 213800_at |
| CFH | I | ENSG00000000971 | 215388_s_at |
| CFH | II | ENSG00000000971 | 215388_s_at |
| CHI3L1 | II | ENSG00000133048 | 209396_s_at |
| CLEC1B | I | ENSG00000165682 | 220496_at |
| CNTD2 | II | ENSG00000105219 | 220323_at |
| CNTNAP2 | II | ENSG00000174469 | 219300_s_at |
| COL4A3 | II | ENSG00000169031 | 216898_s_at |
| CRYAB | I | ENSG00000109846 | 209283_at |
| CSF2 | II | ENSG00000164400 | 210228_at |
| CSTF2T | II | ENSG00000177613 | 212901_s_at |
| CTSZ | II | ENSG00000101160 | 212562_s_at |
| CUL9 | I | ENSG00000112659 | 213204_at |
| CYLD | II | ENSG00000083799 | 39582_at |
| CYLD | II | ENSG00000083799 | 222142_at |
| DDX58 | II | ENSG00000107201 | 218943_s_at |
| DDX58 | I | ENSG00000107201 | 218943_s_at |
| DDX60 | I | ENSG00000137628 | 218986_s_at |
| DDX60 | II | ENSG00000137628 | 218986_s_at |
| DEPTOR | II | ENSG00000155792 | 218858_at |
| DIDO1 | I | ENSG00000101191 | 218325_s_at |
| DOCK9 | II | ENSG00000088387 | 215237_at |
| DOK3 | I | ENSG00000146094 | 220320_at |
| DRAM1 | II | ENSG00000136048 | 218627_at |
| DSCAM | II | ENSG00000171587 | 211484_s_at |
| DUOX2 | I | ENSG00000140279 | 219727_at |
| EPHX2 | II | ENSG00000120915 | 209368_at |
| ERAP1 | II | ENSG00000164307 | 210385_s_at |
| ERAP1 | II | ENSG00000164307 | 214012_at |
| ERAP1 | II | ENSG00000164307 | 209788_s_at |
| ERP44 | II | ENSG00000023318 | 208958_at |
| EVC | I | ENSG00000072840 | 210887_s_at |
| FAM172A | II | ENSG00000113391 | 212936_at |
| FAM204A | II | ENSG00000165669 | 218390_s_at |
| FAM46C | I | ENSG00000183508 | 220306_at |
| FUT4 | I | ENSG00000196371 | 209893_s_at |
| FYN | II | ENSG00000010810 | 212486_s_at |
| FZD10 | II | ENSG00000111432 | 219764_at |
| FZD10 | I | ENSG00000111432 | 219764_at |
| GATA3 | II | ENSG00000107485 | 209602_s_at |
| GDF2 | II | ENSG00000128802 | 221136_at |
| GDPD5 | II | ENSG00000158555 | 213343_s_at |
| GKN1 | I | ENSG00000169605 | 220191_at |
| GMIP | I | ENSG00000089639 | 218913_s_at |
| GOLGA8A | II | ENSG00000175265 | 208797_s_at |
| GPR98 | II | ENSG00000164199 | 215396_at |
| GREM1 | II | ENSG00000166923 | 218468_s_at |
| GSTK1 | II | ENSG00000197448 | 217751_at |
| GYG2 | II | ENSG00000056998 | 215695_s_at |
| HBS1L | II | ENSG00000112339 | 209315_at |
| HEG1 | II | ENSG00000173706 | 213069_at |
| HELLS | I | ENSG00000119969 | 220085_at |
| HERC5 | I | ENSG00000138646 | 219863_at |
| HLA-A | I | ENSG00000206503 | 213932_x_at |
| HLA-A | II | ENSG00000206503 | 215313_x_at |
| HLA-A | I | ENSG00000206503 | 215313_x_at |
| HLA-A | II | ENSG00000206503 | 213932_x_at |
| HLA-C | I | ENSG00000237022 | 208812_x_at |
| HLA-C | II | ENSG00000206435 | 211799_x_at |
| HLA-C | I | ENSG00000233841 | 211799_x_at |
| HLA-C | I | ENSG00000204525 | 216526_x_at |
| HLA-C | II | ENSG00000228299 | 208812_x_at |
| HLA-C | I | ENSG00000206452 | 208812_x_at |
| HLA-C | II | ENSG00000204525 | 216526_x_at |
| HLA-C | II | ENSG00000237022 | 211799_x_at |
| HLA-C | I | ENSG00000225691 | 211799_x_at |
| HLA-C | I | ENSG00000228299 | 211799_x_at |
| HLA-C | II | ENSG00000206435 | 208812_x_at |
| HLA-C | I | ENSG00000225691 | 208812_x_at |
| HLA-C | II | ENSG00000206452 | 211799_x_at |
| HLA-C | I | ENSG00000206435 | 211799_x_at |
| HLA-C | II | ENSG00000237022 | 208812_x_at |
| HLA-C | II | ENSG00000204525 | 211799_x_at |
| HLA-C | II | ENSG00000225691 | 214459_x_at |
| HLA-C | I | ENSG00000237022 | 211799_x_at |
| HLA-C | II | ENSG00000206452 | 208812_x_at |
| HLA-C | II | ENSG00000233841 | 211799_x_at |
| HLA-C | I | ENSG00000228299 | 208812_x_at |
| HLA-C | I | ENSG00000206452 | 211799_x_at |
| HLA-C | II | ENSG00000225691 | 208812_x_at |
| HLA-C | II | ENSG00000225691 | 211799_x_at |
| HLA-C | I | ENSG00000206435 | 208812_x_at |
| HLA-C | II | ENSG00000228299 | 211799_x_at |
| HLA-C | I | ENSG00000225691 | 214459_x_at |
| HLA-C | I | ENSG00000204525 | 211799_x_at |
| HLA-G | II | ENSG00000204632 | 211528_x_at |
| HLA-G | I | ENSG00000204632 | 211530_x_at |
| HLA-G | II | ENSG00000204632 | 211529_x_at |
| HLA-G | II | ENSG00000204632 | 210514_x_at |
| HLA-G | II | ENSG00000204632 | 211530_x_at |
| HLA-G | I | ENSG00000204632 | 211528_x_at |
| HLA-G | I | ENSG00000204632 | 211529_x_at |
| HLA-G | I | ENSG00000204632 | 210514_x_at |
| HLA-J | I | ENSG00000204622 | 217436_x_at |
| HLA-J | II | ENSG00000204622 | 217436_x_at |
| HSPA2 | II | ENSG00000126803 | 211538_s_at |
| ID4 | II | ENSG00000172201 | 209291_at |
| IDO1 | II | ENSG00000131203 | 210029_at |
| IFI35 | I | ENSG00000068079 | 209417_s_at |
| IFI35 | II | ENSG00000068079 | 209417_s_at |
| IFI44 | II | ENSG00000137965 | 214453_s_at |
| IFI44 | I | ENSG00000137965 | 214453_s_at |
| IFIT2 | II | ENSG00000119922 | 217502_at |
| IFITM1 | II | ENSG00000185885 | 214022_s_at |
| IFITM1 | I | ENSG00000185885 | 214022_s_at |
| IFITM1 | I | ENSG00000185885 | 201601_x_at |
| IFITM1 | II | ENSG00000185885 | 201601_x_at |
| IFITM3 | II | ENSG00000142089 | 212203_x_at |
| IFITM3 | I | ENSG00000142089 | 212203_x_at |
| IFNA21 | II | ENSG00000137080 | 211145_x_at |
| IGFLR1 | II | ENSG00000126246 | 219690_at |
| KCNG1 | I | ENSG00000026559 | 214595_at |
| KDM8 | II | ENSG00000155666 | 220070_at |
| KIF1C | II | ENSG00000129250 | 209245_s_at |
| KIF1C | I | ENSG00000129250 | 209245_s_at |
| KIR2DL4 | I | ENSG00000189013 | 208426_x_at |
| KLF2 | II | ENSG00000127528 | 219371_s_at |
| KLF4 | II | ENSG00000136826 | 221841_s_at |
| KLF4 | II | ENSG00000136826 | 220266_s_at |
| LAP3 | II | ENSG00000002549 | 217933_s_at |
| LAP3 | I | ENSG00000002549 | 217933_s_at |
| LRP5L | II | ENSG00000100068 | 214873_at |
| LXN | II | ENSG00000079257 | 218729_at |
| MAGEC2 | II | ENSG00000046774 | 220062_s_at |
| MAP7D3 | II | ENSG00000129680 | 219576_at |
| MEGF9 | II | ENSG00000106780 | 212830_at |
| MLL2 | II | ENSG00000167548 | 216382_s_at |
| MMP26 | I | ENSG00000167346 | 220541_at |
| MT1P2 | II | ENSG00000244020 | 211456_x_at |
| NADK | II | ENSG00000008130 | 208917_x_at |
| NCAM1 | II | ENSG00000149294 | 212843_at |
| NCAM1 | I | ENSG00000149294 | 212843_at |
| NIPAL2 | II | ENSG00000104361 | 220128_s_at |
| NIPAL2 | I | ENSG00000104361 | 220128_s_at |
| NOP14-AS1 | II | ENSG00000249673 | 214685_at |
| NPAT | II | ENSG00000149308 | 211584_s_at |
| OAS3 | II | ENSG00000111331 | 218400_at |
| OAS3 | I | ENSG00000111331 | 218400_at |
| OLFML2A | II | ENSG00000185585 | 213075_at |
| PACRG | II | ENSG00000112530 | 215472_at |
| PACSIN3 | II | ENSG00000165912 | 218744_s_at |
| PADI3 | II | ENSG00000142619 | 220779_at |
| PARP12 | I | ENSG00000059378 | 218543_s_at |
| PARP12 | II | ENSG00000059378 | 218543_s_at |
| PARP3 | II | ENSG00000041880 | 209940_at |
| PCDHA10 | II | ENSG00000250120 | 211867_s_at |
| PCDHA10 | I | ENSG00000250120 | 211867_s_at |
| PDLIM3 | I | ENSG00000154553 | 209621_s_at |
| PDZD8 | II | ENSG00000165650 | 213549_at |
| PHLDA2 | I | ENSG00000181649 | 209802_at |
| PLA1A | II | ENSG00000144837 | 219584_at |
| PLAUR | II | ENSG00000011422 | 210845_s_at |
| PLXNB1 | II | ENSG00000164050 | 215807_s_at |
| PPAP2B | II | ENSG00000162407 | 209355_s_at |
| PRDM1 | II | ENSG00000057657 | 217192_s_at |
| PRRG3 | I | ENSG00000130032 | 220433_at |
| PSMB8 | I | ENSG00000204264 | 209040_s_at |
| PSMB8 | II | ENSG00000204264 | 209040_s_at |
| PYGO1 | I | ENSG00000171016 | 215517_at |
| RCAN3 | II | ENSG00000117602 | 219864_s_at |
| RNF19B | II | ENSG00000116514 | 36564_at |
| RNF19B | II | ENSG00000116514 | 213038_at |
| RNFT1 | II | ENSG00000189050 | 221195_at |
| RTP4 | II | ENSG00000136514 | 219684_at |
| SAMD9 | I | ENSG00000205413 | 219691_at |
| SECTM1 | II | ENSG00000141574 | 213716_s_at |
| SECTM1 | I | ENSG00000141574 | 213716_s_at |
| SELPLG | II | ENSG00000110876 | 209879_at |
| SERGEF | II | ENSG00000129158 | 220482_s_at |
| SLC12A4 | I | ENSG00000124067 | 211112_at |
| SLC15A3 | II | ENSG00000110446 | 219593_at |
| SLC29A3 | II | ENSG00000198246 | 219344_at |
| SOCS1 | II | ENSG00000185338 | 210001_s_at |
| SOCS1 | I | ENSG00000185338 | 209999_x_at |
| SORBS1 | I | ENSG00000095637 | 218087_s_at |
| SQRDL | II | ENSG00000137767 | 217995_at |
| SST | I | ENSG00000157005 | 213921_at |
| STAT3 | II | ENSG00000168610 | 208992_s_at |
| STYK1 | II | ENSG00000060140 | 221696_s_at |
| TAPBPL | II | ENSG00000139192 | 218746_at |
| TAPBPL | II | ENSG00000139192 | 218747_s_at |
| TBC1D13 | I | ENSG00000107021 | 218596_at |
| TDRD7 | I | ENSG00000196116 | 213361_at |
| TLN2 | II | ENSG00000171914 | 212703_at |
| TM2D1 | II | ENSG00000162604 | 213882_at |
| TMEM140 | II | ENSG00000146859 | 218999_at |
| TMOD2 | II | ENSG00000128872 | 219701_at |
| TOP1 | II | ENSG00000198900 | 208900_s_at |
| TRANK1 | I | ENSG00000168016 | 213261_at |
| TRANK1 | II | ENSG00000168016 | 213261_at |
| TRIM22 | I | ENSG00000132274 | 213293_s_at |
| TRIM22 | II | ENSG00000132274 | 213293_s_at |
| TRIM5 | I | ENSG00000132256 | 210705_s_at |
| TRIM62 | II | ENSG00000116525 | 58308_at |
| TUBA1A | II | ENSG00000167552 | 209118_s_at |
| UCP2 | II | ENSG00000175567 | 208998_at |
| UNC93A | I | ENSG00000112494 | 214382_at |
| USP18 | I | ENSG00000184979 | 219211_at |
| WDR25 | II | ENSG00000176473 | 219609_at |
| ZC3HAV1 | I | ENSG00000105939 | 220104_at |
| ZC3HAV1 | II | ENSG00000105939 | 220104_at |
| ZFR2 | II | ENSG00000105278 | 215419_at |
| ZNF287 | I | ENSG00000141040 | 216710_x_at |
| ZNF3 | II | ENSG00000166526 | 219604_s_at |
| ZNF688 | II | ENSG00000229809 | 213529_at |
| ZNF701 | II | ENSG00000167562 | 220242_x_at |
| ZNF804A | II | ENSG00000170396 | 215767_at |
| ABHD6 | I | ENSG00000163686 | 221678_at |
| ABHD6 | II | ENSG00000163686 | 221678_at |
| ARHGEF26 | II | ENSG00000114790 | 222121_at |
| ARHGEF26 | I | ENSG00000114790 | 222121_at |
| EGOT | I | ENSG00000235947 | 222314_x_at |
| GDNF | I | ENSG00000168621 | 221359_at |
| LONP2 | I | ENSG00000102910 | 221833_at |
| LRTM1 | II | ENSG00000144771 | 91580_at |
| LRTM1 | I | ENSG00000144771 | 91580_at |
| MICALL1 | II | ENSG00000100139 | 221779_at |
| OR1G1 | II | ENSG00000183024 | 221375_at |
| PHF11 | I | ENSG00000136147 | 221816_s_at |
| PHF11 | II | ENSG00000136147 | 221816_s_at |
| SLC25A28 | II | ENSG00000155287 | 221432_s_at |
| TRABD | I | ENSG00000170638 | 221807_s_at |
| ZDHHC8P1 | I | ENSG00000133519 | 222274_at |
| ZNF335 | I | ENSG00000198026 | 221890_at |
